# Propensity score adjustment using machine learning classification algorithms to control selection bias in online surveys

**DOI:** 10.1371/journal.pone.0231500

**Published:** 2020-04-22

**Authors:** Ramón Ferri-García, María del Mar Rueda

**Affiliations:** Department of Statistics and Operations Research, Faculty of Sciences, University of Granada, Granada, Spain; University of Pisa, ITALY

## Abstract

Modern survey methods may be subject to non-observable bias, from various sources. Among online surveys, for example, selection bias is prevalent, due to the sampling mechanism commonly used, whereby participants self-select from a subgroup whose characteristics differ from those of the target population. Several techniques have been proposed to tackle this issue. One such is Propensity Score Adjustment (PSA), which is widely used and has been analysed in various studies. The usual method of estimating the propensity score is logistic regression, which requires a reference probability sample in addition to the online nonprobability sample. The predicted propensities can be used for reweighting using various estimators. However, in the online survey context, there are alternatives that might outperform logistic regression regarding propensity estimation. The aim of the present study is to determine the efficiency of some of these alternatives, involving Machine Learning (ML) classification algorithms. PSA is applied in two simulation scenarios, representing situations commonly found in online surveys, using logistic regression and ML models for propensity estimation. The results obtained show that ML algorithms remove selection bias more effectively than logistic regression when used for PSA, but that their efficacy depends largely on the selection mechanism employed and the dimensionality of the data.

## Introduction

One of the main drawbacks of online surveys is the selection bias [1] that may be introduced in their use. This problem occurs when the population sample used differs from the non-observed population in such a way that the sample results cannot be extrapolated to the full population. In online surveys, samples are often drawn from volunteer participants, for reasons of time and financial economy, making this population nonprobabilistic and therefore unsuitable for the usual sampling methods employed for inference and estimation. Assuming that some groups are more likely than others to participate, volunteer samples present an inherent selection bias. Hence, determining optimum probabilistic sampling conditions in an online survey is not a trivial undertaking. As [2] state, probabilistic online frames can only be used when the population of interest is narrow (the members of well-defined organizations); evidently, if the target population is not properly defined a reliable sampling frame of internet users may not be achieved. Internet access is often associated with sociodemographic variables related to the variables of interest in a given study ([3]). For example, according to [4], the internet penetration rate in Spain is above 90% of the population in all population groups aged under 54 years; however, among persons aged 65 to 74 years, the penetration rate is only 43.7%. In consequence, the potentially covered population (as defined in [1]) is immediately subjected to a selection bias, which cannot be completely excluded by the usual reweighting methods ([5]; [6]).

In recent years, propensity score adjustment (PSA) has increasingly been used as a means of correcting selection bias in online surveys. This method, first proposed by [7], was originally intended to correct the bias introduced by factors associated with exposure (group allocation) and outcome in the experimental design, and studies have demonstrated its effectiveness in this regard ([8]; [9]). PSA, like most adjustment instruments in population sampling, is based on the use of auxiliary information. However, in addition to the nonprobabilistic volunteer sample, it also requires the availability of a probabilistic reference sample. This is usually obtained from a survey focused on a different subject area. Accordingly, it does not measure the present variable or variables of interest, but rather a set of covariates that have also been recorded or the nonprobabilistic sample. The reference survey does not have to address the same research questions, but it should be well conducted and avoid all sources of bias as much as possible.

The efficacy of PSA at removing selection bias from online surveys has been discussed in numerous studies. However, its performance depends on the covariates chosen. Moreover, the use of PSA generally increases the sampling variability of the estimators with respect to the unweighted case ([10]; [11]). Therefore, PSA weighting should be complemented with further calibration adjustments using complementary variables to make estimates less biased ([11]; [12]).

Propensity scores in PSA are usually estimated using logistic regression models, where the target variable is a binary indicator that takes 1 if an individual belongs to the nonprobabilistic sample and 0 otherwise. This approach is equivalent to estimating the probability of an individual volunteering to participate in a survey, given a specific set of covariates. Logistic regression provides estimates that are robust, i.e. they remain stable when new data are incorporated, and simple to implement in most statistical packages. However, they also present certain drawbacks that should be taken into account. Thus, in logistic modelling it is assumed that the log-odds risks have a linear relationship with the covariates ([13]). In the online survey context, this assumption could easily fail to hold, especially with larger samples and a greater number of covariates.

Alternatives to logistic regression in PSA have appeared in parallel with the development of machine learning (ML) classification algorithms. A vast and still-increasing number of ML approaches provide the raw probabilities of occurrence of a given class, both black-box and interpretable, the application of which in PSA has mainly been studied with respect to experimental design. Research into interpretable algorithms for PSA has focused on classification and regression trees (CART) ([14]), concluding that these decision trees provide less biased effect estimates, even under conditions of non-additivity and non-linearity ([15]; [16]). In this respect, [17] examined a special case of discriminant analysis, selecting the best classification tree in terms of optimality.

Among the black-box alternatives that have been developed in the field of ML, neural networks and bagging/boosting algorithms have attracted much attention. Neural networks are discussed in [18] as a potential replacement for logistic regression in PSA, but to our knowledge, they have only been successfully applied in [15]. In addition, bagging algorithms such as Random Forest ([19]) and boosting algorithms such as the Gradient Boosting Machine (GBM) ([20]) have been included in several studies. It has been suggested that the use of GBM could provide more stable weights and greater bias reduction than is the case with logit models ([21]) and multinomial models ([22]), at the cost of a minimal increase in variance. Similarly, the incorporation of Random Forest into PSA may also reduce the level of bias in the estimates obtained, compared to logistic models ([16] [23]) and classification trees ([24]). GBM has been successfully applied in real experiments with propensity scores ([25], [26], [27]). In this field, too, [28] studied the performance of boosted CART (GBM), but found that the performance of each algorithm was strongly dependent on the scenario. Finally, [29] analysed the efficiency of propensity estimation, using Random Forests for matching, taking into account the existence of missing data from the predictors, and reported good results for group balancing.

An interesting case of black-box algorithms is described by [30]. These authors used the Super Learner paradigm proposed by [31], and estimated propensity scores by choosing the best algorithm, in terms of goodness-of-fit, from a set of ML classification algorithms, including Bayesian Generalised Linear Models, Support Vector Machines, Multivariate Adaptative Regression Splines and k-Nearest Neighbours, apart from those mentioned above. This study showed that overall efficiency was dependent on the underlying covariate structure, but that PSA, using the Super Learner strategy, presented good balancing properties.

In recent survey research, ML algorithms have been widely studied in the probability sampling context ([32]; [33]; [34]; [35]; [36]). In nonprobability sampling, however, PSA has mainly been used in nonresponse propensity adjustments. The PSA procedure for addressing the question of nonresponse bias, which was first developed by [37], usually follows the same steps as in dealing with selection bias, but some alternatives to logistic regression have been proposed. Thus, [38] used local polynomial regression models to adjust nonresponse propensity estimates, in a paper extending their previous work on propensity estimates via kernel regression. Further details of this method are discussed by [39]. These models provide better estimates of propensity, in terms of likelihood, and lower variance than is the case with logistic regression models, provided that the polynomial degree is properly specified. Applications of ML algorithms in PSA for nonresponse propensity have been studied for classification and regression trees ([40]) and Random Forests ([41]); their ability to reduce nonresponse bias, in comparison with logistic regression, depends on the covariates available and on the complexity of the relationships. These techniques for modelling nonresponse propensity are also addressed by [42].

In the present paper, the ML approach is extended to the question of reducing selection bias, considering various online survey scenarios that are subject to selection bias and examining how PSA may reduce this bias, according to the algorithm used to compute the propensity estimates. The study method and the ML methods used are described in detail, after which we present a simulation study based on artificial and real-world data. The implications of these results are then discussed, and in the final section we suggest related lines of work for future research.

## Propensity Score Adjustment (PSA) for volunteer online samples

The procedure to perform Propensity Score Adjustment for removing volunteer bias in online surveys can be described as follows: let *s*_*v*_ be a volunteer nonprobabilistic sample of size *n*_*vs*_, self-selected from an online population *U*_*v*_ which is a subset of the total target population *U*, and *s*_*r*_ a reference probabilistic sample of size *n*_*rs*_ selected from *U* under a sampling design (*s*_*d*_, *p*_*d*_) with *π*_*i*_ = ∑_*s*_*r*_∋*i*_ *p*_*d*_(*s*_*r*_) the first order inclusion probability for the *i*-th individual. Note that each element in both samples has a base weight associated, say $d_{j}^{v}$, *j* = 1, …, *n*_*vs*_ for the volunteer sample and $d_{k}^{r}$, *k* = 1, …, *n*_*rs*_ for the reference sample (usually, $d_{k}^{r}=1/\pi_{k}$). Covariates **X** used to adjust the propensity scores have been measured on both samples, while the variable of interest *y* has been measured only in the volunteer sample, and the probabilistic sample cannot be directly used for its estimation as a result. Let *z* be a binary variable which measures whether a participant of the complete sample *s* = *s*_*r*_ ∪ *s*_*v*_ belongs to *s*_*r*_ or *s*_*v*_.

$$\begin{array}{c} z_{i}=\{\begin{array}{cc} 0 & i\in s_{r}\\ 1 & i\in s_{v} \end{array},\;\;\;i=1,\ldots ,n,n=n_{vs}+n_{rs} \end{array}$$ (1)

Let *π*(**x**_*i*_) be the propensity score for participant *i* conditional on his/her covariates’ value **x**_*i*_. *π*(**x**_*i*_) reflects the probability of *z*_*i*_ = 1 given the set of covariates **X**. The reference sample is assumed to suffer from a small selection bias or no bias at all, and can be used to generate a reliable estimate of the covariates’ distribution in the target population. This information could be used to calculate which types of individuals are more or less prone to participate in an online survey. The above-mentioned propensity scores, $\hat{\pi }\left(x_{i}\right)$, are often estimated using a logistic regression model which can be described as in Eq 2.

$$\begin{array}{c} \hat{\pi }\left(x_{i}\right)=\frac{1}{e^{-(\gamma^{T}x_{i})}+1} \end{array}$$ (2)

where *γ* is the vector of regression coefficients obtained in the modelling process. The original online sample is reweighted using the propensity estimates to take into account the information on selection bias provided by PSA. This procedure can be performed using weights for either the Horvitz-Thompson or the Hajek estimators; the procedure for the Horvitz-Thompson-type weights is described in [10] and [11] and can be summarised as follows. The combined sample is sorted and then divided into *C* classes ([43] recommend the use of five classes) according to each individual’s propensity score. An appropriate adjustment factor *f*_*c*_ is obtained using Eq 3

$$\begin{array}{c} f_{c}=\frac{\sum_{k\in s_{r}^{c}}d_{k}^{r}/\sum_{k\in s_{r}}d_{k}^{r}}{\sum_{j\in s_{v}^{c}}d_{j}^{v}/\sum_{j\in s_{v}}d_{j}^{v}} \end{array}$$ (3)

where $s_{r}^{c}$ and $s_{v}^{c}$ are individuals from the reference sample and the volunteer sample respectively, belonging to the *c*-th class. The new weights *w* for individuals in the volunteer sample are then calculated as follows:

$$\begin{array}{c} w_{j}=f_{c}d_{j}^{v}=\frac{\sum_{k\in s_{r}^{c}}d_{k}^{r}/\sum_{k\in s_{r}}d_{k}^{r}}{\sum_{j\in s_{v}^{c}}d_{j}^{v}/\sum_{j\in s_{v}}d_{j}^{v}}d_{j}^{v} \end{array}$$ (4)

Hajek-type weights can be calculated as described in [2] and according to Eq 5. In this case, the weights adjust the volunteer sample to the population of the probabilistic sample, *U*_*r*_, rather than the complete population *U*.

$$\begin{array}{c} w_{j}=\frac{1-\hat{\pi }\left(x_{j}\right)}{\hat{\pi }\left(x_{j}\right)} \end{array}$$ (5)

According to [44], the difference between the two approaches to the final estimates depends both on the discreteness of the support of the covariates and on the selection mechanism used (i.e., whether or not it is related to the target variable).

## Machine learning classification algorithms for propensity score estimation

As described above, various alternatives to logistic regression can be used in propensity score estimation, leading to different formulas to obtain $\hat{\pi }\left(x_{i}\right)$. In this section, we present some formulas associated with the application of some algorithms commonly used in PSA literature, together with other techniques frequently seen in data mining ([45]), namely decision trees, Random Forests, GBM, k-Nearest Neighbours and Naïve Bayes.

Decision trees can be defined as a set of rules organised in a hierarchical structure, starting from an initial node that represents the complete dataset. To predict a given individual, the dataset is split into different subsets according to a rule based on an input predictor variable. Each subset can also be split, successively, until a convergence criterion is met; then, the rule stops increasing in complexity, and a terminal node is reached. Any input individual for the decision tree will meet the criteria of a rule specified for it, and thus predicted according to data from the individuals meeting the rule criteria. In our study, the algorithms used for tree building involve C4.5, C5.0 ([46]) and CART ([14]). They differ in some aspects of tree building, such as the rule pruning and complexity, but for brevity these questions are not addressed in the present paper.

This approach can be used to obtain the probabilities of the input individuals of a decision tree belonging to any given class. In this context, these probabilities represent the individuals’ propensity to participate in an online survey, where *z* represents the binary target variable. Let *J*_1_, …, *J*_*k*_ be the set of rules (terminal nodes) of a decision tree; each rule represents a multidimensional range for each covariate, say: *J*_*i*_ = {**X** ∈ *B*_*i*_} where $B_{i}\in R^{p}$, and where *p* is the number of covariates. Let $n(s_{v}^{J_{i}})$ and $n(s^{J_{i}})$ be the number of volunteer sample and combined sample members, respectively, which meet the criteria of the *i*th terminal node. The formula for estimating propensity scores for an individual *i* using decision trees is described in Eq 6.

$$\begin{array}{c} \hat{\pi }\left(x_{i}\right)=\{\begin{array}{cc} \frac{n(s_{v}^{J_{1}})}{n(s^{J_{1}})} & \left\{i\in s/x_{i}\in J_{1}\right\}\\ \ldots & \ldots \\ \frac{n(s_{v}^{J_{k}})}{n(s^{J_{k}})} & \left\{i\in s/x_{i}\in J_{k}\right\} \end{array} \end{array}$$ (6)

In the case of Random Forests, propensities are estimated by averaging the number of times that an input individual is classified in the class representing the presence (often denoted as “1”) through a set of *m* trees known as *weak classifiers*. Input variables for each tree are randomly selected, in subsets of fixed size, from the available covariates. Therefore, the propensity score estimation can be reformulated as in Eq 7.

$$\begin{array}{c} \hat{\pi }\left(x_{i}\right)=\frac{\sum_{j=1}^{m}\phi_{j}\left(x_{i}\right)}{m},\;\phi_{j}\left(x_{i}\right)=\{\begin{array}{cc} 1 & \left\{i\in s/x_{i}\in J_{pr}\right\}\\ 0 & \left\{i\in s/x_{i}\in J_{ab}\right\} \end{array} \end{array}$$ (7)

where *J*_*ab*_ and *J*_*pr*_ represent the set of terminal nodes where individuals from the volunteer sample are minority and majority, respectively. In other words:

$$\begin{array}{c} J_{pr}=\left\{J_{l},l=1,\ldots ,k:\frac{n(s_{v}^{J_{l}})}{n(s^{J_{l}})}\geq 0.5\right\} \end{array}$$ (8)

$$\begin{array}{c} J_{ab}=\left\{J_{l},l=1,\ldots ,k:\frac{n(s_{v}^{J_{l}})}{n(s^{J_{l}})}<0.5\right\} \end{array}$$ (9)

Note that in the cases where the volunteer and the reference sample are very unbalanced in size, the propensity scores may be exactly zero or one for some individuals and in such cases cannot be properly applied. In some studies, adjustments have been made in order to avoid this situation; for instance, [41] applied a (1000 ⋅ *x* + 0.5)/1001 transformation to move the propensities away from zero and one.

For *k* nearest neighbours, computing the propensity score estimates involves a distance function *d* which measures the closeness of each data point to a given individual *i* using covariates **X**. This distance allows the *n* − 1 individuals to be rearranged as **x**_(1)_, …, **x**_(*n*−1)_, where **x**_(1)_ and **x**_(*n*−1)_ represent, respectively, the covariates of the closest and the furthest individual from *i* according to *d*. As the target variable is binary, the propensity scores can be estimated with the following formula:

$$\begin{array}{c} \hat{\pi }\left(x_{i}\right)=\frac{\sum_{j\in s/d\left(x_{i},x_{j}\right)\leq d\left(x_{i},x_{\left(k\right)}\right)}z_{j}}{k} \end{array}$$ (10)

Application of the formula shown in Eq 10 is equivalent to calculating the proportion of individuals from the volunteer sample out of the *k* nearest neighbours to the individual *i*. The number of neighbours *k* is arbitrary, meaning that *k* = 1 or even a small enough *k* will provide probabilities of zero or one.

Estimation of the propensity scores using the Naïve Bayes algorithm is based on the Bayes formula, derived from the observed probabilities of participants belonging to the volunteer sample and the occurrence of a given vector for **X**, that is, the values of the covariates for a given individual *i*.

$$\begin{array}{c} \hat{\pi }\left(x_{i}\right)=\frac{P\left(z_{i}=1\right)P\left(X=x_{i}|z_{i}=1\right)}{P(X=x_{i})} \end{array}$$ (11)

If variables with very rare classes or presenting high cardinality are used as covariates, the propensity estimates might present values significantly far from the real propensity.

Finally, when using a GBM algorithm ([19]), the formula for propensity score estimation has the same structure as that used in logistic regression, but is based on a different parametrisation:

$$\begin{array}{c} \hat{\pi }\left(x_{i}\right)=\frac{1}{e^{-w^{T}J\left(x_{i}\right)}+1} \end{array}$$ (12)

where *J*(**x**_*i*_) represents a matrix of terminal nodes of *m* decision trees (the number of trees used is decided by the user but should be correlated with the sample size, as in Random Forests) and *w* is a vector representing the weights of each tree. The development of trees in *J*(**x**_*i*_) is achieved through an iterative process minimising of the specified loss function for a small sample of the input dataset (which, in this context, is assumed to be the combined sample *s*) selected for testing purposes.

## Simulation study

### Artificial data

To evaluate the performance of classification algorithms applied under different circumstances for PSA, we conducted an experiment using a fictitious population of voters. This population was used originally by [44], following an experiment by [5] to measure the efficiency of adjustments in selection bias. For the present study, minor changes were made to the gender distributions by age so that a proper Missing Completely At Random (MCAR) situation could be simulated. This population and the experiment are detailed below.

The fictitious population had a total size of N = 50,000 individuals. The study aim was to estimate the fraction of votes obtained by each of three fictitious parties, Party 1, 2 and 3, in a hypothetical election. Four sociodemographic variables—age, nationality, gender and education—were measured in each sample and used as covariates for the PSA models.

- The age variable was determined by applying the following transformation of a simulated Beta distribution: *Age* = 82*x* + 18, *x* ∼ *β*(2, 3). The resulting age pyramid was similar to the real-world case in Spain ([47]).
- In the study population, 15% were non-natives aged under 35 years, 10% were non-natives aged 35 to 65 years, and 2.5% were non-natives aged over 65 years, which is similar to the nationality distribution by ages in Spain ([48]).
- The probability of the individual being male or female was identical, for the whole population, in contrast to the experiment performed by [44].
- Education was constrained to be dependent on the age strata (the same strata as for the Nationality variable), in order to make it similar to the pattern of education levels among Spanish adults ([49]).
  - Of the individuals aged under 35 years, 35% had only primary education, 20% had secondary education and 45% had higher education.
  - Of the individuals aged 35 to 65 years, 45% had only primary education, 25% had secondary education and 30% had higher education.
  - Of the individuals aged over 65 years, 80% had only primary education, 10% had secondary education and 10% had higher education.

In addition, internet access was made dependent on age and nationality. Among non-natives, internet access was available to 20% of those aged under 35 years, but only to 10% of those aged 35 to 65 years and to 0% of those aged over 65 years. In contrast, for natives the corresponding values were 90%, 70% and 50% respectively.

The probabilities of a person voting for Party 1, 2 or 3 were considered in relation to the above variables. Party 1 would attract the votes of 20% of the female population, but the men would not vote for it at all. As internet access did not depend on gender, measuring the proportions of the population who would vote for Party 1 could be considered an example of a Missing Completely at Random (MCAR) selection mechanism. For Party 2, the voting probability increased in line with the voter’s age; among the population as a whole, nobody aged under 35 years would vote for this party, while 40% of those aged 35 to 65 years would do so, as would 60% of those over 65 years old. The above-mentioned relationship between age and internet access means that the measurement of voting intentions for Party 2 is also subject to a Missing At Random (MAR) selection mechanism. Finally, voting intentions for Party 3 depended on both age and internet access: thus, 10% of individuals with no internet access (regardless of their age) would vote for this party, while among those with internet access, the party would attract the votes of 60% of those under 35 years, 40% of those aged 35 to 65 years, and 20% of those aged over 65 years. These relationships mean that the measurement of voting intentions for Party 3 is subject to a Not Missing At Random (NMAR) selection mechanism, as the target variable is in fact related to the selection variable.

The distribution of values for the population as a whole and for each of the subpopulations, with and without access to internet, is shown in S1 Table. As expected, there is a slight divergence in voting intentions for Party 2 between the population as a whole and those with internet, and a strong divergence in this respect for Party 3. Persons with internet were more likely to have completed a course of higher education, were on average two years younger and were five times less likely to be non-native. However, differences in gender were negligible, and so voting intentions for Party 1 were barely affected.

To estimate voting percentages for each party, we selected a convenience sample from the population with internet access, and a reference sample from the full population. The reference sample was drawn by simple random sampling without replacement (SRSWOR), and three different sampling schemes were tested to select the convenience sample:

1. SRSWOR from the whole internet population.
2. Sampling from the whole internet population with unequal self-selection probabilities, obtained by the following formula:
  $$\begin{array}{c} \pi_{i}=\frac{1}{1+e^{-1+0.05\cdot {\text{Age}}_{i}}},i=1,2,\ldots ,31,881 \end{array}$$ (13)
  where Age_*i*_ is the age of the *i*-th individual of the internet population.
3. Sampling from the whole internet population with unequal self-selection probabilities, obtained by the following formula:
  $$\begin{array}{c} \pi_{i}=\frac{1}{1+e^{1-sin({\text{Age}}_{i}/20)}},i=1,2,\ldots ,31,881 \end{array}$$ (14)
  where Age_*i*_ is the age of the *i*-th individual of the internet population.

The formulas for the inclusion probabilities in schemes 2 and 3 were tested to evaluate how ML algorithms perform in comparison with logistic regression when the relationship between the covariates and the selection probability (which we assume to be the self-selection probability) can be modelled using the logit function, with either linear or nonlinear relationships. The experiment was replicated varying the convenience sample size across *n*_*vs*_ = 500, 750, 1,000, 2,000, 5,000, 7,500 and 10,000, and the size of the reference survey was established at 500 individuals for each replication. The replication results were obtained by averaging the bias and calculating the MSE of the estimates in 500 simulations. The mean bias of each replication was obtained according to Eq 15:

$$\begin{array}{c} {\text{Bias}}_{k}=\frac{\sum_{m=1}^{500}{\hat{p}}_{m}^{k}}{500}-p^{k} \end{array}$$ (15)

where ${\hat{p}}_{m}^{k}$ is the proportion of voters for Party *k* estimated in the *m*-th simulation and *p*^*k*^ is the real proportion of voters for Party *k*. The MSE for the estimators in each replication was obtained directly from the estimates, as in Eq 16:

$$\begin{array}{c} {\text{MSE}}_{k}=\frac{\sum_{m=1}^{500}{\left({\hat{p}}_{m}^{k}-{\hat{\bar{p}}}^{k}\right)}^{2}}{499}+{\left({\text{Bias}}_{k}\right)}^{2} \end{array}$$ (16)

where ${\hat{\bar{p}}}^{k}$ is the mean of the estimates for the proportion of voters for Party *k*.

### Real data

A set of real data was analysed to determine the usual patterns observed in real applications. This real-data approach is commonly employed in studies of PSA ([10]; [11]; [12]).

The dataset used to simulate a pseudo-population was obtained from the microdata of the 2012 edition of the Life Conditions Survey (known by the Spanish acronym, ECV) ([50]). This annual survey is conducted face-to-face by the Spanish Institute of Statistics, targeting the entire Spanish population aged 16 years or older. The primary unit considered is the household, and the secondary units are the members of the household. The variables considered include income, poverty, equality, employment and household living conditions. In 2012, 12,714 households were surveyed, providing a sample population of 33,573 individuals. In this study, the full sample had to be preprocessed before the simulation due to the considerable volume of missing data. After this filtering process, the size of the pseudo-population (that is, the full filtered dataset) was N = 28,210, and 61 variables were identified as potential covariates for the PSA models.

The convenience and reference samples were selected by SRSWOR from the volunteer (internet) population and the full population, respectively. The identifying variable for volunteers and non-volunteers was the presence of a computer in the household. According to the 2012 Spanish Survey on Equipment and Use of Information and Communication Technologies in Households ([51]), 90.1% of persons who had a computer in their household also had internet access, and 98.3% of those with internet access at home also had a computer. Therefore, we believe it reasonable to assume that taking the presence of a computer in the household as the selection variable is a very good proxy of a variable measuring internet access in the household. Two target variables were considered:

- The proportion of the population whose self-reported health was poor (those who responded “poor” or “very poor” to the question regarding their general state of health.
- The proportion of the population living in a household with more than two members.

The experiment was replicated 500 times, varying the size of the convenience sample across *n*_*vs*_ = 500, 750, 1,000, 2,000, 5,000 while the reference sample size was maintained at *n*_*rs*_ = 500, and considering the following groups of covariates:

- Group 1: Nine covariates measuring region, size of home town/city, gender, marital status, nationality, country of origin and education level (both achieved and currently studying).
- Group 2: All the covariates in Group 1 plus five health-related variables, namely chronic diseases, presence of disability, and lack of access to medical and/or dentistry services (and reasons for this lack).
- Group 3: All the covariates in Group 1 plus eleven poverty-related variables, namely delays in bill payment, incidence of bills on the household economy, difficulty in living within household income, ability to acquire certain household goods, possession of electrical appliances, income needed to live without financial difficulty and calculated indicators of poverty risk and material scarcity.
- Group 4: All 61 potential covariates. All of the above variables plus working conditions, care provision, energy poverty and household conditions and expenditure.

The S1 Dataset includes the full dataset used to perform these analyses.

### Algorithms and parameter tuning

The procedure in both simulations was the same: in each of the 500 simulations, convenience and reference samples were selected, PSA was applied to reweight the convenience sample using Hajek-type weights, and the population parameter was estimated using the convenience sample with PSA. Measures of bias and MSE for each scenario, algorithm and *n*_*vs*_ were estimated as in ]^18^] and [14]. This procedure was implemented in the statistical software R ([52]) using the packages *RWeka* ([53]; [54]),*C50* ([55]), *rpart* ([56]), *randomForest* ([57]), *e1071* ([58]) abd *gbm* ([59]). Packages *ggplot2* ([60]), *xlsx* ([61]), *gridExtra* ([62]) and *RColorBrewer* ([63]) were used to generate the figures illustrating the results.

Propensity scores were calculated in each case using logistic regression, C4.5, C5.0, CART, k-nearest neighbors, Naïve Bayes, Random Forest, and GBM. For exploratory purposes, in the artificial data simulation the parameter configuration of each algorithm was selected on a grid, as follows:

- Decision trees (C4.5, C5.0 and CART) were applied taking 0.1, 0.25 and 0.5 as confidence values for pruning, and 0.5%, 1% and 5% of the dataset as the minimum number of observations per node.
- K-Nearest Neighbours was applied taking *k* = 3, 5, 7, 9, 11, 13.
- Naïve Bayes was applied with a slight Laplace smoothing for the values 0, 1, 2, 5, 10.
- Random Forests were generated with 500 trees and 1, 2, 4 sampled variables for each tree.
- GBM was applied with interaction depths of 4, 6 and 8, and learning rates of 0.1, 0.01 and 0.001.

The impact of tuning parameters in PSA is still poorly understood, and optimality criteria are lacking. In this context, goal of classification algorithms is not to achieve greater accuracy but a higher likelihood for the propensity of volunteer participation in an online survey ([11]).

Parameter tuning was implemented for real data simulation. Thus, 10 times repeated 10-fold cross-validation was performed for each scenario, algorithm and *n*_*vs*_ using the *caret* package in R ([64]), except for the CART algorithm, for which the cross-validation was coded separately, as *caret* does not allow us to refine the minimum number of observations per node. Log-Loss optimisation was used, as this metric bettter explains the deviation of estimated propensities from real participation. The parameter grids were as described above, with the following exceptions: the sampled variables for the Random Forest trees were taken as $\sqrt{p}$, *p*/2 and *p*, where *p* is the number of covariates. In C5.0, we did not optimise the confidence value for pruning and minimum number of observations in the nodes. The optimal values obtained for C4.5 were used in C5.0, because the two algorithms are closely related and likely to behave in a similar way. On the other hand, the trials, type of model (rule-based or tree-based) and winnowing (feature selection) were tuned in C5.0. The results obtained are summarised in S2 Table.

## Results

### Artificial data

S3 and S4 Tables show the bias and MSE results, respectively, obtained from using PSA with ML algorithms and SRSWOR from the internet population to build the convenience sample. There are small differences in bias reduction between C4.5, C5.0 and CART, especially for larger volunteer sample sizes. For Party 1, these algorithms outperform logistic regression only when the volunteer sample size is small, converging to the unadjusted case for larger samples. For Parties 2 and 3, the three algorithms are only better than unadjusted estimations when the sample sizes are balanced, but they never improve on PSA estimates using logistic regression. The MSE estimators with C4.5, C5.0 and CART also converge to the unadjusted case, which is smaller than PSA with logistic regression for Party 1 but greater for Parties 2 and 3. The parameter tuning of decision trees (with any algorithm: CART, C4.5 or C5.0) has no significant effect on bias removal, although greater confidence in the pruning appears to be slightly advantageous in the case of Parties 2 and 3 if the C4.5 algorithm is used and the sample sizes are relatively balanced.

The use of the k-NN classifier yields less biased estimates than that of baseline logistic regression for all of the missing data mechanisms considered. Thus, PSA with k-NN provides estimates that are less biased, on average, than the unadjusted estimates and the default-PSA reweighted estimates for Party 3. For Party 2, reweighting with PSA using k-NN transforms the bias in the opposite direction to the original bias; however, this bias is lower than that produced by the PSA estimates with logistic regression in absolute terms. For Party 1, k-NN provides less biased estimates than logistic regression but a low value for *k*; moreover, larger sample sizes are required. When estimating the likelihood of an individual voting for Party 1 or 2, the estimator MSE is smaller when PSA is used with k-NN rather than logistic regression, for larger numbers of neighbours and balanced sample sizes. When this is done for Party 3, the MSE of PSA with k-NN is significantly lower than for PSA with logistic regression, although large values of *k* are required for full efficiency.

Application of the Naïve Bayes classifier in PSA produces a substantially greater reduction in bias than when PSA is performed with logistic regression, but only for the case of Party 2. For the other two parties, Naïve Bayes does not outperform logistic regression in PSA in terms of estimation bias except when samples are balanced and larger integers are used for Laplace smoothing. In addition, the MSE of the estimators is smaller with Naïve Bayes when the sample sizes are balanced and Laplace smoothing uses larger integers. The improvement, however, is rather limited.

Propensity estimation with the Random Forest algorithm is only advantageous in terms of bias removal in the estimations for Party 3, in which case the Random Forests algorithm achieves the highest bias reduction of all the classifiers reviewed. This is an important finding, as this missing data mechanism is particularly troublesome and, moreover, is commonly encountered in real data. The results for the MSE estimators under PSA with Random Forests show that this value may be only half that obtained with PSA and logistic regression for Party 3. The number of candidate variables for tree growing provides better results, remaining low for balanced sample sizes, but high for larger samples.

Finally, the efficiency of PSA reweighting with GBM is crucially dependent on the parameter configuration employed. For all kinds of missing data mechanisms, PSA with GBM removes bias more effectively when the learning rate is relatively low; thus, for Party 2, the bias reduction is almost complete. The MSE of the estimators reveal that GBM for PSA is advantageous for Parties 1 and 2 if parameter fitting is adequate (lower learning rates for Party 1, higher ones for Party 2), and significantly advantageous for Party 3 when the learning rate is high. The effects of interaction depth are mainly apparent with larger volunteer sample sizes, and greater interaction depths provide estimations with lower levels of bias and MSE.

S5 and S6 Tables show the results obtained from using PSA with ML algorithms with unequal selection probabilities in the internet population, following the logistic formula described in Eq 13 for convenience sampling, for bias and MSE, respectively. As in the previous scenario, bias reduction with PSA using decision trees (C4.5, C5.0 or CART) converges to the unadjusted case as the convenience sample size increases. The best results are provided by C4.5 trees, but these are still much worse than those obtained with logistic regression. Regarding MSE, the lack of variability produced by the inability of decision trees to grow in samples with a large fraction of volunteer respondents leads them to have a smaller error than is the case with logistic regression in the estimation of intentions to vote for Party 1, especially with CART. Parameter tuning has a noticeable (albeit small) effect only when the samples are relatively balanced in size: with C4.5, higher confidence in pruning leads to better results, while with CART the opposite is true.

Using the k-NN algorithm in PSA produces a greater bias reduction than that of logistic regression for Parties 2 and 3, provided the number of neighbours, *k*, and the sample size are sufficiently large. The increase in variability provoked by the use of this algorithm makes the MSE slightly higher than with logistic regression in the intention to vote for Party 2. However, this is not the case regarding Party 3, where PSA with k-NN provides estimates with less error. In the case of Naïve Bayes, and regardless of the Laplace smoothing used, the bias and MSE are greater for Party 2 than with logistic regression, but these values are smaller for Party 3. Comparatively, Naïve Bayes in PSA provides estimates which produce a smaller error than either logistic regression, decision trees or k-NN.

The bias removal provided by PSA with Random Forest is strongly dependent on the size of the convenience sample and the number of variables sampled to create the trees. The bias for Party 3 is close to zero when the convenience sample is around four times larger than the reference sample and only two variables are sampled. If four variables are sampled, the bias reduction is greatest when the sample is 10 to 15 times greater. These results show that the Random Forest algorithm again provides the best MSE results in estimating voting probabilities for Party 3.

The GBM algorithm applied in PSA for sampling with unequal selection probabilities produces a very similar situation to SRSWOR, except that efficiency decreases in line with the size of the convenience sample size. When comparing the MSE in the voting estimation for Party 2 with that of k-NN and logistic regression, GBM is poorer with small sample sizes but better with larger ones. Accordingly, GBM is the best option for estimating voting intentions for Party 2 when a large convenience sample is available.

S7 and S8 Tables show the results for unequal selection probabilities in the internet population following the logistic formula described in Eq 14 for convenience sampling, for bias and MSE, respectively. The performance of all the algorithms, taking into account that the amount of inherent bias is smaller, is very similar to the previous case. Among the differences observed, it should be noted that bias reduction is worse with k-NN (especially in estimating voting intentions for Party 2, for which this algorithm performs no better than logistic regression) and that Naïve Bayes performs better for Party 2 but worse for Party 3.

S9 Table summarises the bias and MSE measures obtained for each algorithm and selection mechanism, revealing certain characteristic patterns. For Party 1, while Naïve Bayes provides the lowest mean bias and is the best adjustment more frequently than the other algorithms, decision trees are better choices in terms of MSE, especially CART. For Party 2, bias reduction is dominated by PSA with k-NN and GBM but the former is surpassed by GLM in terms of MSE. Finally, Random Forest seems to be the best algorithm for PSA regarding voting intentions for Party 3, both in terms of bias and MSE. In general, ML algorithms (except for decision trees) produce the largest reductions in bias and, in many cases too, the lowest MSE.

### Real data

S10 Table show the results obtained for the bias present in estimating the fraction of the population who perceive their health to be poor. The table rows show the estimations obtained after PSA reweighting with the four covariate groups. These results clearly reflect the importance of the variables regarding PSA efficiency; when only demographic variables are included, PSA with Naïve Bayes provides the least biased estimates for all sample sizes, but also greater variance than the other methods and hence a larger MSE. In consequence, PSA with logistic regression provides the smallest error term. The bias is smaller when variables related to the outcome (health) or the exposure (poverty) are included in the models, with the former group leading to greater reductions in bias and MSE, but in this respect the situation for algorithms is unaffected. However, when all available covariates are used, PSA with Naïve Bayes appears to produce high levels of bias, while PSA with logistic regression is almost unbiased for large volunteer sample sizes, at the cost of high variance. As a result, MSE values are poor for PSA with logistic regression, while decision trees (for small *n*_*vs*_) and bagging/boosting algorithms (for large *n*_*vs*_) have the smallest term of error. The estimation with the lowest MSE was achieved using PSA with logistic regression together with demographic and health-related predictors, followed by PSA with GBM using all available predictors.

S11 Table shows the bias estimates for the fraction of households with more than two members, after PSA reweighting. It is noticeable that PSA with Random Forest removes most of the original bias as the volunteer sample size increases when only demographic variables are used, to the point that the MSE of the estimates obtained by PSA with Random Forest with *n*_*vs*_ is the lowest of all those observed during the experiment. This pattern continues when health-related variables are added, although the bias of the estimates increases. On the other hand, if small volunteer samples are used, PSA with logistic regression provides the estimates with the smallest error term, and this true for all sample sizes when poverty covariates are used. When all covariates are included, a similar pattern is observed: thus, decision trees and GBM (with the latter providing the second-lowest MSE of the experiment), are the best algorithms for PSA when small and large sample sizes, respectively, are available.

## Discussion

New technologies have had a profound impact on surveying techniques worldwide. This impact is especially significant for social and political surveys, and most particularly for market research surveys, where the speed increases and cost reductions achieved with new technologies have radically changed the ways in which data are compiled. While in many cases the public sector continues to conduct interviews face-to-face and/or via telephone landlines, private companies are using mobile phones, tablets and the web, as standalone or combined strategies, thus obtaining data from volunteer participants. On the other hand, the results obtained with such nonprobability surveys present various problematic issues, notably the absence of a sample design assigning weights to the sample units, the presence of frame coverage issues and the risk of nonresponse bias. Although many statistical methods have been proposed to alleviate the problems of noncoverage and nonresponse, the question of nonrandomness in the sample is more complex and has not been thoroughly addressed.

In this respect, [65] reviewed existing inference methods to correct for selection bias in nonprobability samples. These authors considered a situation where only a nonprobability sample is available and compared a range of predictive inference methods (pseudo-design-based and model-based) in a general framework. The conclusion drawn from this study was that machine learning methods should be incorporated to address the problem of misrepresentation in nonprobability samples.

The present study considers another class of methods that may be used to correct selection bias in volunteer online surveys, which combine a nonprobability sample with a reference sample in order to construct propensity models. Our analysis compares logistic regression and ML classification algorithms for propensity estimation to determine the extent to which ML may be considered a viable alternative. ML algorithms present certain advantages over logistic regression; for example, they present greater flexibility, and do not require the analyst to specify a model with its interactions on nonlinear relationships, as ML is capable of capturing these relationships in the data learning procedure. Our study considers situations with few and with many covariates, for three different missing-data mechanisms influencing the selection process, and for different parameter configurations in the classifiers. To our knowledge, the only previous studies of the efficiency of classifier parameter tuning in PSA are those of [15] and [16] for decision trees, and to a more limited extent than in the present case. In addition, [41] alluded to some preliminary tests for Random Forest parameters, suggesting that optimum parameter selection would improve the estimations achieved.

The results we present show that most of the algorithms evaluated may provide a valid alternative to logistic regression in PSA if circumstances make the latter inappropriate. The C4.5 and C5.0 algorithms for decision trees are particularly useful when reference and volunteer sample sizes are balanced and the variables are numerous. Decision trees can be considered as variable selectors, as they automatically select subsets of optimal variables for classification, which is advantageous when the dimensionality is high ([66]). However, they also increase estimation variance when used for PSA, especially when there are significant nonlinear relationships between variables and the sample size is small ([16]; [28]).

The k-Nearest Neighbours (k-NN) algorithm is another useful alternative to logistic regression in PSA if the number of covariates available is low, especially with NMAR selection. However, as the dimensionality increases, k-NN becomes less efficient than other approaches. Its behaviour in both low and high-dimensional contexts was studied by [67], who concluded that higher dimensionality results in more concentrated distances, which makes k-NN less explicative of the actual class of an individual.

Our evaluation of Naïve Bayes in PSA for controlling selection bias revealed the existence of certain very clear patterns. When used with balanced sample sizes and few covariates, and not presenting rare or infrequent values, this algorithm provides smaller MSE values. In any other case, although PSA with Naïve Bayes behaves in an unstable way, simulations for NMAR using real data show that MSE is also substantially reduced. Ideally, Naïve Bayes should be employed with discrete input variables, as the probability computation performed by the algorithm is based on cross-tabulations. In addition, Naïve Bayes assumes independence between the variables, which may not be realistic in a high dimensional context due to the redundancy and noise issues that often arise (see [66]).

The application of bagging and boosting algorithms produced interesting results. Random Forest, which has been widely tested for PSA ([16]; [23]; [22]; [24]; [30]; [41]; [29]), achieved the largest bias reduction when the selection mechanism was NMAR, both for simulated data (under the condition of sample balancing) and with real data. However, its application presented several drawbacks, especially the fact that it is very prone to overfit propensity estimates on the data, as was apparent in the MSE of the Random Forest estimates with PSA, which tended to decrease and stabilise as the volunteer sample size increased. This pattern of behaviour has been reported previously by [24] for treatment effect estimates, and by [41], who observed an increase in variance when Random Forests of classification trees were used. On the other hand, the GBM, also referred to in the literature as boosted CART ([16]; [30]), provided weights that resulted in more stable behaviour of the estimates, as has also been noted previously ([16]; [22]). The GBM is efficient if the parameters are correctly tuned and the covariates are sufficiently discriminant. In this respect, [21] proposed a default parameter configuration for the GBM with low interaction depth and shrinkage. In the simulated data example described, better results were obtained with greater interaction depths. On the other hand, the best results with artificial data simulation were obtained when the learning rate was maximal; this parameter is related to overfit, and therefore should not produce a different pattern of behaviour in other situations. Nonetheless, further research is needed on the question of GBM parameter fitting. Finally, let us note that PSA with GBM in the real data simulation provided the best results in terms of MSE, for a large volunteer sample and when all available covariates were used.

## Conclusion

Our study findings support the use of ML algorithms as an alternative to PSA for reducing or eliminating selection bias in online surveys, although logistic regression is also shown to be a robust, reliable technique for propensity estimation. The efficiency of ML algorithms is closely related to the type of data considered and therefore no single approach is optimum for every case. We provide evidence with respect to MCAR, MAR and NMAR selection mechanisms, and for situations of low or high dimensionality. When selection follows a MCAR scheme, CART and GBM are the best alternatives, although the other ML algorithms tested, except Random Forest, also improve upon the results obtained by PSA with logistic regression, especially as the volunteer sample size increases. With MAR or NMAR selection, logistic regression generally provides good adjustments, especially when the dimensionality is low and the covariates are not very discriminant. However, if more covariates are available, logistic regression tends to destabilise and the MSE increases, despite its improved performance in bias removal; in this case, GBM, k-NN, decision trees and Random Forests all represent good alternatives. Random Forests provides good results when the data are MCAR, even if covariates are nonsignificant, although more research is needed on the possible incidence of overfitting on the final results obtained. The presence of balancing and overfitting issues suggests that data preprocessing should be a key step in the estimation of propensity scores, as observed previously by [17]. We recommend that further studies should consider the application of data preprocessing techniques such as noise filtering, sample balancing or feature selection (see [68]) before PSA application, and also take into account the effects of dimensionality when designing simulation experiments or applications.

In general, our findings support the view given in [65] that ML methods can usefully be used to remove selection bias when dealing with non-probability samples. Prior research has shown that PSA successfully removes bias in some situations but at the cost of increasing the variance of the estimates ([10]; [11]). The technique proposed by [11] and [12], applying a combination of PSA and calibration, may represent a good alternative in such situations. The behaviour of ML methods when both PSA and calibration are applied is currently under study.

## Supporting information

S1 Table Summary statistics of the simulated population and the subpopulations with and without internet access. (PDF)

S2 Table Optimal parameters for each algorithm given a volunteer sample size and a group of covariates in the real data simulation, obtained with a 10 times repeated 10-fold cross-validation. (PDF)

S3 Table Bias on the estimation of vote intention with unequal selection probabilities for the convenience sample based on SRSWOR from the internet population. (PDF)

S4 Table Mean Square Error (MSE) in the estimation of vote intention with unequal selection probabilities for the convenience sample based on SRSWOR from the internet population. (PDF)

S5 Table Bias in the estimation of vote intention with unequal selection probabilities for the convenience sample based on the logistic formula. (PDF)

S6 Table Mean Square Error (MSE) in the estimation of vote intention with unequal selection probabilities for the convenience sample based on the logistic formula. (PDF)

S7 Table Bias in the estimation of vote intention with unequal selection probabilities for the convenience sample based on the logistic formula with a sine transformation. (PDF)

S8 Table Mean Square Error (MSE) in the estimation of vote intention with unequal selection probabilities for the convenience sample based on the logistic formula with a sine transformation. (PDF)

S9 Table Mean and median of bias (absolute values) and MSE of estimates using PSA for each algorithm, and number of times its estimates are among the best (absolute bias or MSE less than 1% greater than the minimum value). (PDF)

S10 Table Bias and MSE prevalence estimates of self-reported “poor health” status after reweighting with PSA using classification algorithms. (PDF)

S11 Table Bias and MSE of the estimates of the fraction of households with more than two members after reweighting with PSA using classification algorithms. (PDF)

S1 Dataset Datasets used in the simulation study. (DOCX)
