# Supplementary material for: Propensity score adjustment using machine learning classification algorithms to control selection bias in online surveys
Source: PLoS One. 2020 Apr 22;15(4):e0231500. doi: 10.1371/journal.pone.0231500 (PMC7176094; doi:10.1371/journal.pone.0231500)
Supplement: S1 Table — (PDF) [file pone.0231500.s001.pdf]

S1 Table. Summary statistics of the simulated population and the subpopulations with and without Internet access.

Variable	Stats / Values	Full population (N = 50,000)	Internet population (N = 31,881)	No Internet population (N = 18,119)
Vote	Party 1	10.2%	10.0%	10.6%
	Party 2	36.7%	33.8%	41.7%
	Party 3	29.9%	41.3%	9.8%
Education	Primary	50.6%	48.3%	54.6%
	Secondary	21.1%	21.5%	20.4%
	Tertiary	28.3%	30.2%	25.1%
Age	Mean (s. d.)	50.8 (16.5)	48.5 (16.1)	54.8 (16.4)
	Median	50	47	54
Gender	Man	50.2%	50.6%	49.7%
	Woman	49.8%	49.4%	50.3%
Nationality	Native	90.9%	98.2%	77.9%
	Non-native	9.1%	1.8%	22.1%
