# Supplementary material for: Propensity score adjustment using machine learning classification algorithms to control selection bias in online surveys
Source: PLoS One. 2020 Apr 22;15(4):e0231500. doi: 10.1371/journal.pone.0231500 (PMC7176094; doi:10.1371/journal.pone.0231500)
Supplement: S2 Table — (PDF) [file pone.0231500.s002.pdf]

S2 Table. Optimal parameters for each algorithm given a volunteer sample size and a group of covariates in the real data simulation, obtained with a 10 times repeated 10-fold cross-validation

		KNN	C5.0			CART	
n_{vs}	Cov.	k	Trials	Model	Winnow	CP	% sample
500	G1	11	1	tree	TRUE	0.1	0.5%
500	G2	11	1	tree	TRUE	0.1	0.5%
500	G3	11	1	tree	FALSE	0.1	0.5%
500	G4	11	1	tree	TRUE	0.1	0.5%
750	G1	11	1	tree	TRUE	0.1	0.5%
750	G2	11	1	tree	TRUE	0.5	0.5%
750	G3	11	1	tree	FALSE	0.25	1%
750	G4	11	1	tree	TRUE	0.1	5%
1000	G1	11	1	tree	TRUE	0.5	0.5%
1000	G2	11	1	tree	FALSE	0.25	5%
1000	G3	11	1	tree	FALSE	0.5	5%
1000	G4	11	1	tree	TRUE	0.25	5%
2000	G1	11	1	tree	TRUE	0.25	1%
2000	G2	11	1	tree	TRUE	0.25	1%
2000	G3	11	1	tree	FALSE	0.5	0.5%
2000	G4	11	1	tree	TRUE	0.5	0.5%
5000	G1	11	1	tree	TRUE	0.1	1%
5000	G2	11	1	tree	TRUE	0.1	1%
5000	G3	11	1	tree	FALSE	0.25	1%
5000	G4	11	1	tree	TRUE	0.5	0.5%
		C4.5		Naive Bayes	Random Forest	GBM	
n_{vs}	Cov. group	CP	% sample	Laplace	Vars. in trees	ID	LR
500	G1	0.1	0.5%	0	p	8	0.01
500	G2	0.1	1%	0	p	8	0.01
500	G3	0.1	1%	0	$\sqrt{p}$	8	0.01
500	G4	0.1	1%	0	$p/2$	8	0.01
750	G1	0.1	0.5%	0	p	8	0.01
750	G2	0.5	1%	0	p	8	0.01
750	G3	0.5	5%	0	$p/2$	4	0.1
750	G4	0.5	5%	0	$p/2$	8	0.01
1000	G1	0.5	0.5%	0	p	6	0.01
1000	G2	0.5	0.5%	0	p	6	0.01
1000	G3	0.25	1%	0	$p/2$	4	0.1
1000	G4	0.1	1%	0	$\sqrt{p}$	8	0.01
2000	G1	0.1	0.5%	0	p	6	0.01
2000	G2	0.1	0.5%	0	p	4	0.01
2000	G3	0.5	1%	0	$p/2$	8	0.01
2000	G4	0.5	1%	0	$\sqrt{p}$	8	0.01
5000	G1	0.1	0.5%	0	p	8	0.01
5000	G2	0.1	0.5%	0	p	4	0.01
5000	G3	0.5	0.5%	0	$p/2$	4	0.1
5000	G4	0.5	0.5%	0	$\sqrt{p}$	4	0.1
