# Supplementary material for: Propensity score adjustment using machine learning classification algorithms to control selection bias in online surveys
Source: PLoS One. 2020 Apr 22;15(4):e0231500. doi: 10.1371/journal.pone.0231500 (PMC7176094; doi:10.1371/journal.pone.0231500)
Supplement: S3 Table — (PDF) [file pone.0231500.s003.pdf]

S3 Table. Bias on the estimation of vote intention with unequal selection probabilities for the convenience sample based on SRSWOR from the Internet population

			Convenience (Internet) sample sizes, in thousands of respondents																				
			Estimates for Party 1							Estimates for Party 2							Estimates for Party 3						
Method	Parameters		0.5	0.75	1	2	5	7.5	10	0.5	0.75	1	2	5	7.5	10	0.5	0.75	1	2	5	7.5	10
No adjustment			-0.2	-0.1	-0.2	-0.2	-0.2	-0.2	-0.2	-2.7	-2.9	-2.7	-2.8	-2.9	-2.8	-2.9	11.3	11.4	11.4	11.4	11.4	11.4	11.4
Logistic regression			-0.1	-0.1	-0.2	-0.2	-0.1	-0.1	-0.1	-0.6	-0.7	-0.6	-0.7	-0.7	-0.7	-0.7	9.8	9.9	9.9	9.9	9.8	9.9	9.9
C4.5	M	CP																					
	0.005	0.1	-0.3	-0.1	-0.2	-0.2	-0.2	-0.2	-0.2	-1.6	-3.3	-3.4	-3.3	-2.9	-2.9	-2.9	10.5	11.6	11.7	11.6	11.4	11.4	11.4
	0.005	0.25	-0.2	-0.1	-0.1	-0.2	-0.2	-0.2	-0.2	-1.8	-3.0	-3.3	-3.3	-2.9	-2.9	-2.9	10.6	11.2	11.6	11.8	11.5	11.4	11.4
	0.005	0.5	0.0	-0.1	-0.2	-0.1	-0.2	-0.2	-0.2	-1.5	-2.0	-2.3	-3.2	-3.0	-2.9	-2.9	10.3	10.8	11.0	11.6	11.5	11.4	11.4
	0.01	0.1	-0.1	-0.1	-0.2	-0.2	-0.2	-0.2	-0.2	-1.8	-3.3	-3.4	-3.3	-2.9	-2.8	-2.8	10.5	11.5	11.7	11.7	11.4	11.4	11.4
	0.01	0.25	-0.1	-0.2	-0.2	-0.1	-0.2	-0.2	-0.2	-1.7	-2.9	-3.3	-3.3	-2.9	-2.9	-2.9	10.6	11.4	11.7	11.8	11.4	11.4	11.4
	0.01	0.5	-0.2	-0.2	-0.2	-0.2	-0.2	-0.2	-0.2	-1.3	-2.1	-2.5	-3.3	-2.9	-2.9	-2.9	10.2	10.7	11.2	11.6	11.5	11.4	11.4
	0.05	0.1	-0.2	-0.1	-0.2	-0.2	-0.2	-0.2	-0.2	-1.8	-3.5	-3.6	-2.9	-2.9	-2.8	-2.9	10.4	11.7	11.8	11.4	11.4	11.4	11.4
	0.05	0.25	0.0	-0.1	-0.1	-0.2	-0.2	-0.2	-0.2	-1.7	-3.0	-3.6	-2.9	-2.9	-2.9	-2.9	10.4	11.3	11.7	11.4	11.4	11.5	11.4
	0.05	0.5	0.0	-0.1	-0.1	-0.2	-0.2	-0.2	-0.2	-1.4	-2.6	-3.5	-2.9	-2.8	-2.9	-2.9	10.1	11.0	11.7	11.4	11.4	11.4	11.4
C5.0	M	CP																					
	0.005	0.1	-0.1	-0.3	-0.2	-0.2	-0.2	-0.2	-0.2	-2.3	-3.5	-3.7	-3.1	-2.8	-2.9	-2.9	10.7	11.8	11.9	11.5	11.4	11.4	11.4
	0.005	0.25	-0.1	-0.2	-0.1	-0.2	-0.2	-0.2	-0.2	-2.3	-3.4	-3.6	-3.1	-2.9	-2.8	-2.8	10.8	11.7	11.8	11.5	11.5	11.4	11.4
	0.005	0.5	-0.1	-0.1	-0.1	-0.2	-0.2	-0.2	-0.2	-2.3	-3.6	-3.6	-3.1	-2.9	-2.8	-2.9	11.0	11.8	11.8	11.5	11.4	11.4	11.4
	0.01	0.1	-0.1	-0.1	-0.2	-0.2	-0.2	-0.2	-0.2	-2.4	-3.6	-3.6	-3.1	-2.9	-2.9	-2.9	11.0	11.8	11.9	11.6	11.4	11.4	11.4
	0.01	0.25	-0.2	-0.2	-0.1	-0.2	-0.2	-0.2	-0.2	-2.4	-3.5	-3.6	-3.2	-2.8	-2.9	-2.9	11.0	11.7	11.8	11.5	11.4	11.4	11.4
	0.01	0.5	-0.2	-0.1	-0.2	-0.2	-0.2	-0.2	-0.2	-2.2	-3.5	-3.6	-3.1	-2.9	-2.9	-2.9	10.8	11.7	11.8	11.5	11.4	11.4	11.4
	0.05	0.1	-0.2	-0.1	-0.1	-0.2	-0.2	-0.2	-0.2	-2.5	-3.6	-3.5	-2.9	-2.9	-2.9	-2.9	11.0	11.8	11.7	11.5	11.4	11.4	11.4
	0.05	0.25	-0.2	-0.2	-0.2	-0.2	-0.2	-0.2	-0.2	-2.5	-3.4	-3.4	-2.8	-2.9	-2.8	-2.9	11.0	11.8	11.7	11.3	11.4	11.4	11.4
	0.05	0.5	-0.1	-0.1	-0.2	-0.1	-0.2	-0.2	-0.2	-2.5	-3.5	-3.5	-2.8	-2.9	-2.8	-2.9	11.1	11.7	11.7	11.3	11.4	11.4	11.4
CART	M	CP																					
	0.005	0.1	-0.2	-0.2	-0.2	-0.2	-0.2	-0.2	-0.2	-2.7	-2.8	-2.9	-2.9	-2.9	-2.8	-2.9	11.2	11.3	11.4	11.4	11.4	11.4	11.4
	0.005	0.25	-0.1	-0.2	-0.2	-0.2	-0.2	-0.2	-0.2	-3.0	-2.7	-2.9	-2.8	-2.9	-2.9	-2.9	11.4	11.3	11.5	11.4	11.4	11.4	11.4
	0.005	0.5	-0.1	-0.2	-0.1	-0.2	-0.2	-0.2	-0.2	-2.9	-2.9	-2.9	-2.8	-2.9	-2.8	-2.9	11.4	11.4	11.3	11.3	11.4	11.4	11.4
	0.01	0.1	-0.1	-0.3	-0.2	-0.2	-0.2	-0.2	-0.2	-2.8	-2.9	-2.9	-2.9	-2.9	-2.8	-2.9	11.3	11.5	11.4	11.4	11.4	11.4	11.4
	0.01	0.25	-0.2	-0.2	-0.2	-0.2	-0.2	-0.2	-0.2	-2.8	-2.8	-2.9	-2.9	-2.9	-2.9	-2.8	11.4	11.2	11.5	11.4	11.4	11.4	11.4
	0.01	0.5	-0.1	-0.1	-0.2	-0.2	-0.2	-0.2	-0.2	-2.9	-2.8	-2.9	-2.9	-2.9	-2.9	-2.9	11.3	11.2	11.4	11.4	11.4	11.4	11.4
	0.05	0.1	-0.1	-0.2	-0.2	-0.2	-0.2	-0.2	-0.2	-2.9	-2.8	-2.8	-2.8	-2.8	-2.9	-2.9	11.4	11.4	11.4	11.3	11.4	11.4	11.4
	0.05	0.25	-0.3	-0.2	-0.2	-0.1	-0.2	-0.2	-0.2	-2.6	-2.9	-3.0	-2.9	-2.8	-2.9	-2.9	11.4	11.4	11.5	11.4	11.4	11.4	11.4
	0.05	0.5	-0.3	-0.2	-0.2	-0.2	-0.2	-0.2	-0.2	-2.8	-2.9	-2.8	-2.8	-2.9	-2.9	-2.8	11.4	11.3	11.3	11.4	11.5	11.4	11.4
k-NN	k																						
	3		-0.1	-0.2	-0.1	-0.1	-0.1	-0.1	-0.1	0.0	0.1	0.5	0.6	0.4	0.4	0.2	9.6	9.6	9.1	9.0	9.2	9.1	9.2
	5		-0.3	-0.1	-0.2	-0.2	-0.2	-0.2	-0.1	0.4	0.2	0.5	0.4	0.4	0.4	0.5	9.3	9.2	9.1	9.1	9.2	9.1	9.1
	7		-0.1	-0.2	-0.2	-0.1	-0.1	-0.2	-0.1	0.4	0.5	0.4	0.4	0.4	0.5	0.5	9.3	9.1	9.2	9.2	9.1	9.2	9.1
	9		-0.2	-0.2	-0.1	-0.2	-0.1	-0.2	-0.2	0.5	0.3	0.4	0.5	0.5	0.4	0.5	9.1	9.3	9.2	9.1	9.1	9.2	9.1
	11		-0.2	-0.2	-0.1	-0.2	-0.2	-0.2	-0.2	0.4	0.4	0.4	0.5	0.5	0.4	0.5	9.2	9.3	9.1	9.2	9.1	9.2	9.2
	13		-0.2	-0.2	-0.2	-0.2	-0.1	-0.2	-0.2	0.5	0.5	0.4	0.5	0.4	0.5	0.4	9.2	9.1	9.2	9.1	9.2	9.1	9.1
Naive	laplace																						
Bayes	0		-0.1	0.0	-0.1	0.0	-0.1	-0.1	-0.1	-0.7	-0.9	-0.9	-1.0	-1.0	-1.0	-1.0	9.8	9.9	10.0	10.1	10.0	10.0	10.0
	1		-0.1	0.0	-0.1	-0.1	-0.1	-0.1	-0.1	-0.9	-0.9	-0.8	-0.8	-0.9	-0.9	-1.0	10.0	9.8	9.9	9.9	10.0	10.0	10.0
	2		0.0	-0.1	-0.2	-0.1	-0.1	-0.1	-0.1	-0.8	-1.0	-0.9	-1.1	-1.0	-1.0	-1.0	9.8	10.3	9.9	10.1	10.1	10.0	10.0
	5		-0.1	0.0	-0.2	-0.1	-0.1	-0.1	-0.1	-0.5	-0.9	-0.7	-1.1	-1.0	-1.1	-1.1	9.6	9.9	9.9	10.1	10.0	10.1	10.1
	10		-0.1	-0.1	-0.1	-0.1	-0.1	-0.1	-0.1	-0.8	-0.7	-0.8	-0.9	-1.2	-1.1	-1.2	9.9	9.9	9.9	10.0	10.2	10.2	10.1
Random	mtry																						
Forest	1		0.2	0.2	0.1	0.6	0.7	0.4	0.2	3.5	2.8	-2.1	-6.7	-3.8	-2.5	-2.8	6.8	6.5	9.5	13.0	12.0	11.0	11.3
	2		0.2	0.1	0.1	0.3	0.4	0.4	0.2	4.8	3.5	1.0	-2.4	-2.7	-1.9	-2.6	5.9	6.2	7.7	10.2	10.2	10.1	10.8
	4		-0.6	-0.4	0.2	-0.1	-0.1	-0.1	-0.5	2.2	2.0	1.7	1.9	2.1	1.0	-0.4	8.5	8.5	8.1	7.6	6.3	7.1	8.7
GBM	ID	LR																					
	4	0.1	-0.2	-0.1	-0.1	-0.1	-0.1	-0.1	-0.1	0.1	0.0	0.1	-0.1	0.1	0.2	0.0	9.3	9.4	9.5	9.6	9.4	9.4	9.5
	4	0.01	-0.1	-0.2	-0.1	-0.2	-0.2	-0.2	-0.2	-1.1	-1.2	-1.2	-1.2	-1.5	-1.7	-1.8	10.1	10.4	10.3	10.3	10.4	10.6	10.7
	4	0.001	-0.1	-0.2	-0.2	-0.2	-0.2	-0.2	-0.2	-2.5	-2.6	-2.7	-2.7	-2.7	-2.7	-2.7	11.1	11.2	11.2	11.3	11.3	11.3	11.3
	6	0.1	0.0	0.0	-0.2	-0.1	-0.1	-0.1	-0.1	-0.1	0.1	0.0	0.0	0.0	0.1	0.0	9.5	9.4	9.6	9.5	9.4	9.4	9.5
	6	0.01	-0.2	-0.1	-0.3	-0.1	-0.1	-0.2	-0.2	-1.0	-1.1	-1.1	-1.2	-1.3	-1.4	-1.6	10.2	10.2	10.3	10.2	10.3	10.4	10.5
	6	0.001	-0.2	-0.3	-0.2	-0.2	-0.2	-0.2	-0.2	-2.7	-2.6	-2.7	-2.6	-2.7	-2.7	-2.7	11.2	11.4	11.4	11.2	11.3	11.3	11.3
	8	0.1	-0.3	-0.1	-0.1	-0.1	0.0	-0.1	-0.1	0.1	0.1	0.2	0.1	0.1	0.1	0.1	9.6	9.5	9.3	9.4	9.4	9.4	9.4
	8	0.01	-0.2	-0.1	-0.1	-0.2	-0.1	-0.2	-0.2	-1.0	-0.9	-1.2	-1.3	-1.3	-1.2	-1.3	10.1	10.0	10.3	10.4	10.3	10.3	10.4
	8	0.001	-0.3	-0.1	-0.2	-0.2	-0.2	-0.2	-0.2	-2.6	-2.6	-2.6	-2.6	-2.6	-2.6	-2.6	11.4	11.2	11.2	11.3	11.2	11.2	11.2
