# Supplementary material for: Propensity score adjustment using machine learning classification algorithms to control selection bias in online surveys
Source: PLoS One. 2020 Apr 22;15(4):e0231500. doi: 10.1371/journal.pone.0231500 (PMC7176094; doi:10.1371/journal.pone.0231500)
Supplement: S4 Table — (PDF) [file pone.0231500.s004.pdf]

S4 Table. Mean Square Error (MSE) on the estimation of vote intention with unequal selection probabilities for the convenience sample based on SRSWOR from the Internet population.

			Convenience (Internet) sample sizes, in thousands of respondents																				
			Estimates for Party 1							Estimates for Party 2							Estimates for Party 3						
Method	Parameters		0.5	0.75	1	2	5	7.5	10	0.5	0.75	1	2	5	7.5	10	0.5	0.75	1	2	5	7.5	10
No adjustment			1.9	1.2	0.9	0.5	0.2	0.1	0.1	12.2	11.4	9.1	8.9	8.8	8.3	8.4	131.7	134.0	131.5	130.6	129.9	129.9	130.3
Logistic regression			2.6	1.7	1.3	0.8	0.4	0.3	0.3	7.1	5.3	3.8	2.4	1.5	1.3	1.2	103.2	102.6	101.9	100.7	97.8	98.7	98.6
C4.5	M	CP																					
	0.005	0.1	2.6	1.7	1.3	0.7	0.2	0.1	0.1	8.4	15.6	15.1	12.6	8.7	8.5	8.4	118.3	139.9	140.2	136.8	130.8	130.3	130.3
	0.005	0.25	2.8	1.8	1.4	0.6	0.2	0.1	0.1	10.3	13.6	14.0	12.7	9.0	8.4	8.5	120.1	130.4	138.4	140.0	131.8	130.4	130.1
	0.005	0.5	2.9	1.6	1.4	0.8	0.2	0.1	0.1	10.6	10.0	9.9	12.1	9.5	8.4	8.3	114.1	121.3	124.5	137.3	132.2	130.0	130.2
	0.01	0.1	2.3	1.9	1.2	0.7	0.2	0.1	0.1	11.0	15.1	14.4	12.5	8.7	8.3	8.3	117.7	137.1	140.5	137.9	130.7	129.7	130.5
	0.01	0.25	2.5	1.8	1.2	0.6	0.2	0.1	0.1	10.2	13.6	14.0	12.8	8.7	8.4	8.4	119.1	133.5	139.1	140.1	130.7	129.7	129.6
	0.01	0.5	2.8	1.9	1.5	0.7	0.2	0.1	0.1	9.1	9.6	10.6	12.6	8.6	8.4	8.3	111.9	120.1	128.8	137.4	131.7	129.8	130.0
	0.05	0.1	2.7	1.6	1.2	0.5	0.2	0.1	0.1	9.9	15.8	15.5	9.3	8.6	8.2	8.6	115.9	141.1	142.4	131.2	131.0	130.2	130.6
	0.05	0.25	3.0	1.7	1.2	0.5	0.2	0.1	0.1	10.6	13.6	15.6	9.6	8.7	8.8	8.5	115.6	131.9	141.0	131.2	130.7	131.5	130.8
	0.05	0.5	2.8	1.6	1.3	0.5	0.2	0.1	0.1	8.4	11.9	15.1	9.1	8.4	8.5	8.3	110.9	126.0	140.2	130.9	129.8	129.8	129.5
C5.0	M	CP																					
	0.005	0.1	2.7	1.8	1.1	0.5	0.2	0.1	0.1	12.8	16.1	16.1	10.9	8.2	8.6	8.3	120.7	142.4	144.3	133.2	129.5	130.5	130.5
	0.005	0.25	2.5	1.6	1.2	0.5	0.2	0.1	0.1	12.4	15.6	16.0	11.1	8.9	8.2	8.3	125.3	141.4	142.2	133.6	131.6	129.6	129.8
	0.005	0.5	2.6	1.8	1.3	0.5	0.2	0.1	0.1	12.1	16.5	16.1	11.0	8.6	8.2	8.3	127.8	144.5	142.8	134.7	130.5	130.2	129.2
	0.01	0.1	2.1	1.8	1.1	0.5	0.2	0.1	0.1	13.2	17.2	16.0	10.9	8.6	8.4	8.4	127.5	144.1	144.2	135.3	131.0	129.8	129.8
	0.01	0.25	2.2	1.6	1.2	0.5	0.2	0.1	0.1	12.8	15.5	15.9	11.3	8.4	8.6	8.6	127.8	141.5	141.4	133.9	130.8	131.2	130.9
	0.01	0.5	2.5	1.7	1.2	0.4	0.2	0.1	0.1	11.6	16.6	15.8	10.7	8.9	8.5	8.3	124.6	141.9	143.3	133.2	131.1	131.3	129.9
	0.05	0.1	2.4	1.7	1.0	0.5	0.2	0.1	0.1	12.6	16.9	14.5	9.7	8.6	8.4	8.4	128.2	144.7	138.8	132.3	131.1	129.9	130.1
	0.05	0.25	2.3	1.6	1.1	0.4	0.2	0.1	0.1	13.4	16.0	14.6	9.0	8.8	8.3	8.4	128.6	142.9	140.4	129.7	130.5	129.6	129.9
	0.05	0.5	2.6	1.8	1.1	0.5	0.2	0.1	0.1	14.2	15.7	15.1	9.1	8.5	8.3	8.3	130.3	141.6	141.1	128.2	130.8	130.2	129.6
CART	M	CP																					
	0.005	0.1	2.0	1.2	0.9	0.4	0.2	0.1	0.1	11.9	10.9	10.4	9.5	8.7	8.3	8.4	130.5	131.6	133.1	131.4	130.7	129.7	129.8
	0.005	0.25	1.9	1.3	1.0	0.5	0.2	0.1	0.1	13.3	10.3	10.5	9.0	8.5	8.4	8.4	133.2	130.4	135.5	130.7	130.4	130.6	130.0
	0.005	0.5	1.8	1.3	0.9	0.5	0.2	0.1	0.1	12.6	11.3	10.4	8.8	8.5	8.2	8.5	134.4	132.4	129.6	129.0	130.0	129.2	130.2
	0.01	0.1	1.8	1.2	0.9	0.5	0.2	0.1	0.1	12.5	11.2	10.6	9.4	8.7	8.3	8.3	132.4	134.0	132.7	132.2	130.8	129.6	129.5
	0.01	0.25	2.0	1.3	0.9	0.5	0.2	0.1	0.1	12.3	10.9	10.7	9.5	8.9	8.4	8.2	134.4	129.0	134.0	131.3	130.8	129.3	129.8
	0.01	0.5	1.8	1.1	0.9	0.5	0.2	0.1	0.1	13.0	10.9	10.8	9.4	8.8	8.4	8.3	133.3	129.4	132.1	131.4	130.7	130.4	130.0
	0.05	0.1	1.9	1.3	0.9	0.4	0.2	0.1	0.1	13.4	10.8	10.0	9.0	8.5	8.5	8.3	135.4	134.3	133.4	129.5	130.8	130.9	129.9
	0.05	0.25	1.8	1.3	0.8	0.4	0.2	0.1	0.1	11.5	11.2	11.0	9.5	8.4	8.4	8.3	133.7	133.9	133.6	131.3	129.4	129.8	130.0
	0.05	0.5	1.9	1.3	0.9	0.4	0.2	0.1	0.1	12.4	10.9	9.8	9.0	8.8	8.5	8.2	134.8	131.2	129.0	130.2	132.1	130.9	129.0
k-NN	k																						
	3		4.0	2.3	2.1	1.2	0.6	0.5	0.4	8.7	6.5	5.4	3.2	1.8	1.5	1.2	101.6	98.9	88.4	83.5	85.2	84.2	85.8
	5		2.9	2.1	1.6	1.0	0.6	0.4	0.4	7.7	5.6	5.4	2.8	1.7	1.5	1.4	93.8	90.5	86.5	85.6	85.3	84.2	83.2
	7		2.6	1.9	1.4	0.9	0.4	0.4	0.4	6.8	5.2	3.9	2.6	1.6	1.4	1.4	92.7	87.8	87.3	86.5	84.5	84.7	82.9
	9		2.2	1.6	1.2	0.8	0.4	0.4	0.3	6.5	4.7	4.3	2.7	1.8	1.4	1.3	89.3	91.5	87.6	85.7	84.4	84.6	83.9
	11		2.3	1.6	1.1	0.6	0.5	0.4	0.4	6.0	4.7	3.7	2.6	1.7	1.5	1.3	90.9	90.6	86.4	85.6	83.4	85.0	84.4
	13		2.1	1.5	1.1	0.7	0.4	0.4	0.3	6.2	4.4	3.6	2.6	1.6	1.4	1.3	90.3	85.8	87.6	84.6	84.9	83.7	84.3
Naive	laplace																						
Bayes	0		2.5	1.9	1.5	0.8	0.4	0.3	0.3	7.3	5.1	4.3	3.3	2.3	2.0	2.0	102.9	103.1	103.3	103.1	101.7	101.4	100.5
	1		2.3	1.6	1.3	0.8	0.4	0.3	0.3	7.0	4.7	3.7	2.8	2.2	1.9	1.9	107.5	101.4	100.7	100.5	100.0	100.8	101.1
	2		2.2	1.5	1.3	0.6	0.5	0.3	0.3	6.8	5.3	4.3	3.1	2.4	1.9	1.9	103.0	109.5	101.8	104.3	102.2	101.2	101.3
	5		2.2	1.6	1.3	0.7	0.4	0.3	0.3	5.8	4.5	4.0	3.3	2.2	2.3	2.2	98.0	101.6	100.5	103.7	101.1	101.8	103.1
	10		2.2	1.5	1.2	0.7	0.4	0.3	0.3	6.1	4.3	4.1	3.0	2.6	2.3	2.3	103.4	101.1	101.4	102.5	104.3	103.7	103.4
Random	mtry																						
Forest	1		14.2	11.5	17.3	22.3	10.7	4.8	2.5	25.4	46.2	56.3	90.4	36.2	17.0	13.3	56.8	79.4	145.4	221.1	165.8	130.0	131.5
	2		11.2	6.7	7.6	12.6	12.5	8.9	5.7	41.7	35.8	22.5	35.2	34.4	26.7	23.9	48.8	55.7	80.1	131.9	130.9	123.1	132.1
	4		19.1	10.2	24.8	6.0	10.3	11.8	11.8	87.9	47.1	37.5	21.4	32.3	36.8	30.6	148.1	114.4	93.6	73.3	64.1	78.1	111.4
GBM	ID	LR																					
	4	0.1	2.2	1.9	1.4	0.8	0.4	0.4	0.3	5.8	4.6	3.5	2.1	1.4	1.1	0.9	93.1	93.4	93.3	94.9	89.9	88.6	90.4
	4	0.01	1.9	1.3	1.0	0.5	0.2	0.1	0.1	5.8	4.4	4.0	2.7	2.9	3.2	3.7	106.7	110.7	107.9	106.5	109.6	111.9	114.9
	4	0.001	1.8	1.3	0.9	0.5	0.2	0.1	0.1	10.3	9.4	9.3	8.2	7.6	7.6	7.5	128.4	129.4	128.9	128.2	127.4	128.3	126.8
	6	0.1	2.6	1.9	1.5	0.8	0.5	0.4	0.3	6.9	4.2	4.2	2.2	1.2	1.2	1.1	96.8	92.3	96.0	92.0	89.9	89.0	90.0
	6	0.01	2.1	1.3	1.0	0.4	0.2	0.1	0.1	6.1	4.3	3.3	2.7	2.3	2.4	2.9	109.6	108.4	108.5	106.1	107.5	108.5	111.1
	6	0.001	1.7	1.2	0.9	0.4	0.2	0.1	0.1	11.7	9.6	9.6	8.0	7.6	7.3	7.4	130.7	132.8	131.4	126.6	128.1	126.9	127.2
	8	0.1	2.8	1.9	1.5	0.8	0.5	0.4	0.4	6.7	4.7	4.0	2.4	1.4	1.1	1.1	99.1	94.6	91.0	90.7	88.6	89.6	88.6
	8	0.01	1.8	1.2	0.9	0.5	0.2	0.2	0.1	5.4	3.9	3.7	2.8	2.3	2.0	2.2	106.1	103.2	109.0	109.4	106.8	106.6	107.5
	8	0.001	1.8	1.2	0.8	0.5	0.2	0.1	0.1	11.2	10.0	9.1	7.9	7.1	7.0	7.1	133.7	127.9	128.7	128.4	126.1	126.3	126.4
