# Supplementary material for: Propensity score adjustment using machine learning classification algorithms to control selection bias in online surveys
Source: PLoS One. 2020 Apr 22;15(4):e0231500. doi: 10.1371/journal.pone.0231500 (PMC7176094; doi:10.1371/journal.pone.0231500)
Supplement: S5 Table — (PDF) [file pone.0231500.s005.pdf]

S5 Table. Bias on the estimation of vote intention with unequal selection probabilities for the convenience sample based on the logistic formula.

			Convenience (Internet) sample sizes, in thousands of respondents																				
			Estimates for Party 1							Estimates for Party 2							Estimates for Party 3						
Method	Parameters		0.5	0.75	1	2	5	7.5	10	0.5	0.75	1	2	5	7.5	10	0.5	0.75	1	2	5	7.5	10
No adjustment			-0.1	-0.2	-0.2	-0.2	-0.2	-0.2	-0.2	-12.7	-12.6	-12.6	-12.4	-11.9	-11.4	-10.9	17.4	17.4	17.4	17.3	17.0	16.8	16.5
Logistic regression			0.0	-0.1	-0.2	-0.1	-0.2	-0.1	-0.1	-1.0	-0.9	-0.9	-0.7	-0.5	-0.5	-0.5	10.2	10.2	10.2	10.1	10.0	10.0	9.9
C4.5	M	CP																					
	0.005	0.1	-0.2	-0.2	-0.2	-0.2	-0.2	-0.2	-0.2	-4.5	-4.8	-3.9	-6.1	-11.8	-11.4	-11.0	12.5	12.2	11.5	12.9	17.0	16.7	16.5
	0.005	0.25	-0.2	-0.2	-0.3	-0.2	-0.2	-0.2	-0.2	-3.6	-4.0	-3.6	-4.3	-11.4	-11.4	-11.0	11.9	11.7	11.3	11.9	16.7	16.8	16.5
	0.005	0.5	-0.2	-0.1	-0.1	-0.1	-0.1	-0.2	-0.2	-1.8	-2.3	-3.0	-3.5	-9.5	-11.3	-10.9	11.0	10.7	10.9	11.3	15.3	16.7	16.5
	0.01	0.1	-0.2	-0.2	-0.2	-0.2	-0.2	-0.2	-0.2	-4.6	-4.9	-4.0	-6.4	-11.8	-11.4	-10.9	12.7	12.2	11.5	13.2	17.0	16.8	16.5
	0.01	0.25	0.0	-0.1	-0.1	-0.1	-0.2	-0.2	-0.2	-3.9	-4.5	-3.9	-4.7	-11.7	-11.4	-10.9	12.0	12.0	11.4	11.9	16.9	16.8	16.5
	0.01	0.5	0.0	-0.2	-0.3	0.0	-0.1	-0.2	-0.2	-2.2	-2.5	-3.0	-3.9	-11.2	-11.4	-10.9	10.8	10.7	11.0	11.4	16.5	16.8	16.5
	0.05	0.1	-0.1	-0.2	-0.2	-0.2	-0.2	-0.2	-0.2	-4.6	-5.2	-4.4	-7.3	-11.8	-11.4	-11.0	12.6	12.6	11.8	13.8	17.0	16.8	16.5
	0.05	0.25	-0.2	-0.2	-0.1	-0.2	-0.2	-0.2	-0.2	-4.1	-4.6	-4.4	-5.7	-11.9	-11.4	-10.9	12.4	12.2	11.9	12.6	17.0	16.8	16.5
	0.05	0.5	0.0	-0.1	0.0	-0.2	-0.2	-0.2	-0.2	-2.9	-3.9	-4.2	-4.4	-11.9	-11.4	-11.0	11.5	11.7	11.5	11.7	17.0	16.8	16.5
C5.0	M	CP																					
	0.005	0.1	-0.2	-0.2	-0.2	-0.1	-0.2	-0.2	-0.2	-4.4	-4.6	-4.5	-7.9	-11.8	-11.4	-10.9	12.5	12.3	12.0	14.2	17.0	16.7	16.5
	0.005	0.25	-0.1	-0.2	-0.2	-0.3	-0.2	-0.2	-0.2	-4.0	-4.7	-4.5	-7.5	-11.9	-11.4	-11.0	12.2	12.2	12.0	14.0	17.1	16.8	16.5
	0.005	0.5	-0.2	-0.2	-0.2	-0.1	-0.2	-0.2	-0.2	-4.3	-4.4	-4.6	-7.5	-11.9	-11.4	-10.9	12.4	12.1	12.0	13.9	17.0	16.8	16.5
	0.01	0.1	-0.1	-0.2	-0.1	-0.2	-0.2	-0.2	-0.2	-5.0	-4.7	-4.5	-8.1	-11.9	-11.4	-10.9	12.9	12.2	11.9	14.4	17.0	16.7	16.5
	0.01	0.25	-0.2	-0.2	-0.2	-0.3	-0.2	-0.2	-0.2	-4.2	-4.5	-4.5	-7.8	-11.9	-11.4	-10.9	12.3	12.1	12.1	14.2	17.0	16.8	16.5
	0.01	0.5	-0.2	-0.1	-0.1	-0.2	-0.2	-0.2	-0.2	-4.1	-4.5	-4.6	-7.8	-11.9	-11.4	-10.9	12.3	12.0	12.1	14.0	17.0	16.8	16.5
	0.05	0.1	-0.3	-0.1	-0.2	-0.2	-0.1	-0.2	-0.2	-4.5	-4.7	-4.4	-8.7	-11.9	-11.4	-10.9	12.7	12.3	12.0	14.7	17.0	16.7	16.5
	0.05	0.25	-0.2	-0.1	-0.1	-0.2	-0.2	-0.2	-0.2	-4.2	-4.7	-4.3	-8.6	-11.9	-11.4	-10.9	12.4	12.2	11.9	14.7	17.0	16.7	16.5
	0.05	0.5	-0.2	-0.3	-0.2	-0.2	-0.2	-0.1	-0.2	-4.5	-4.6	-4.8	-8.2	-11.8	-11.5	-10.9	12.5	12.5	12.3	14.4	17.0	16.8	16.5
CART	M	CP																					
	0.005	0.1	-0.2	-0.2	-0.2	-0.2	-0.1	-0.2	-0.2	-4.5	-5.5	-8.7	-12.4	-11.9	-11.4	-10.9	12.6	13.1	14.9	17.3	17.0	16.8	16.5
	0.005	0.25	-0.3	-0.2	-0.2	-0.2	-0.2	-0.2	-0.2	-5.5	-12.5	-12.5	-12.4	-11.9	-11.4	-11.0	13.5	17.4	17.4	17.3	17.0	16.8	16.5
	0.005	0.5	-0.2	-0.2	-0.2	-0.2	-0.2	-0.2	-0.2	-12.7	-12.5	-12.4	-12.5	-11.8	-11.4	-10.9	17.4	17.4	17.3	17.4	17.0	16.8	16.5
	0.01	0.1	-0.2	-0.2	-0.1	-0.2	-0.2	-0.2	-0.2	-4.5	-5.5	-8.9	-12.4	-11.9	-11.4	-10.9	12.8	13.1	15.0	17.3	17.0	16.8	16.5
	0.01	0.25	-0.3	-0.1	-0.2	-0.2	-0.2	-0.2	-0.2	-5.6	-12.5	-12.5	-12.4	-11.8	-11.4	-10.9	13.4	17.2	17.3	17.3	17.0	16.7	16.5
	0.01	0.5	-0.1	-0.1	-0.1	-0.2	-0.2	-0.2	-0.2	-12.5	-12.6	-12.5	-12.3	-11.9	-11.4	-10.9	17.3	17.4	17.3	17.2	17.0	16.7	16.5
	0.05	0.1	-0.2	-0.1	-0.1	-0.2	-0.2	-0.2	-0.2	-4.5	-5.6	-8.5	-12.4	-11.9	-11.4	-10.9	12.8	13.1	14.5	17.3	17.0	16.8	16.5
	0.05	0.25	-0.2	-0.2	-0.2	-0.2	-0.2	-0.2	-0.2	-5.8	-12.5	-12.6	-12.4	-11.9	-11.4	-10.9	13.6	17.3	17.4	17.3	17.0	16.8	16.5
	0.05	0.5	-0.1	-0.1	-0.2	-0.2	-0.2	-0.2	-0.2	-12.6	-12.5	-12.5	-12.3	-11.8	-11.4	-10.9	17.4	17.3	17.4	17.2	17.0	16.8	16.5
k-NN	k																						
	3		-0.2	-0.3	-0.1	-0.1	-0.1	-0.1	-0.1	-2.4	-1.6	-1.0	-0.1	0.1	0.0	-0.1	11.4	10.7	10.5	9.6	9.3	9.4	9.4
	5		-0.2	-0.2	-0.1	-0.2	-0.1	-0.2	-0.2	-1.3	-0.6	-0.6	0.1	0.3	0.4	0.3	10.7	9.9	10.0	9.4	9.2	9.3	9.3
	7		-0.1	-0.2	-0.1	-0.1	-0.2	-0.1	-0.1	-0.7	0.0	0.0	0.5	0.6	0.3	0.3	10.0	9.6	9.5	9.2	9.0	9.2	9.2
	9		-0.2	-0.2	-0.2	-0.2	-0.1	-0.1	-0.1	-0.4	0.1	0.2	0.3	0.5	0.4	0.3	9.7	9.5	9.3	9.3	9.1	9.2	9.2
	11		-0.2	-0.2	-0.1	-0.1	-0.1	-0.1	-0.1	-0.3	0.3	0.4	0.5	0.4	0.5	0.5	9.9	9.3	9.2	9.1	9.1	9.1	9.1
	13		-0.1	-0.1	-0.2	-0.2	-0.2	-0.2	-0.2	-0.3	0.0	0.3	0.3	0.5	0.4	0.3	9.8	9.4	9.2	9.3	9.1	9.1	9.2
Naive	laplace																						
Bayes	0		0.1	0.0	-0.1	-0.3	-0.1	0.0	-0.1	4.7	5.0	5.3	4.9	4.2	3.9	3.4	5.1	5.0	5.2	5.3	5.9	6.0	6.5
	1		0.0	0.0	-0.1	-0.1	-0.1	-0.1	-0.1	4.8	5.0	4.8	4.9	4.3	3.6	3.3	5.2	5.0	5.5	5.2	5.7	6.3	6.5
	2		-0.1	0.1	0.0	-0.3	0.0	-0.1	-0.1	5.1	4.4	4.9	5.0	4.2	3.7	3.3	5.2	5.4	5.1	5.4	5.7	6.2	6.6
	5		-0.1	-0.3	-0.1	0.0	-0.1	0.0	-0.1	5.4	5.1	5.1	4.9	4.2	3.6	3.3	5.0	5.1	5.2	5.1	5.8	6.1	6.6
	10		0.2	-0.2	-0.2	-0.1	-0.1	-0.1	0.0	4.6	5.2	4.7	4.7	4.0	3.6	3.1	5.0	5.1	5.4	5.5	5.9	6.2	6.7
Random	mtry																						
Forest	1		-0.2	0.0	-0.1	0.1	0.2	-0.1	-0.1	3.2	6.3	7.2	8.4	5.8	2.4	-1.2	7.8	5.2	3.8	1.6	4.5	7.5	10.1
	2		0.2	-0.1	-0.3	0.1	0.0	0.1	-0.2	8.2	9.3	11.4	11.4	9.1	6.8	4.7	3.7	2.8	0.8	0.0	1.7	3.5	5.5
	4		-0.3	-0.6	-0.2	0.0	0.7	0.0	-0.1	0.6	3.2	5.2	9.1	11.0	9.8	7.8	9.5	7.5	6.3	2.9	-0.6	0.1	1.9
GBM	ID	LR																					
	4	0.1	0.0	-0.1	-0.2	-0.1	-0.2	-0.1	-0.1	-0.8	-0.4	-0.4	-0.4	-0.2	-0.3	-0.3	10.0	9.7	9.8	9.8	9.6	9.7	9.6
	4	0.01	-0.2	-0.2	-0.2	-0.2	-0.2	-0.2	-0.2	-5.9	-5.6	-5.8	-5.8	-5.6	-5.4	-5.3	13.4	13.0	13.2	13.1	12.9	12.8	12.7
	4	0.001	-0.2	-0.3	-0.2	-0.2	-0.2	-0.2	-0.2	-11.7	-11.6	-11.6	-11.5	-11.1	-10.6	-10.2	16.9	16.8	16.8	16.8	16.5	16.2	16.0
	6	0.1	-0.2	0.0	0.0	-0.2	-0.1	-0.1	-0.1	-1.1	-0.6	-0.5	-0.3	-0.2	-0.1	-0.2	10.4	9.8	9.7	9.8	9.6	9.5	9.5
	6	0.01	0.0	-0.2	-0.2	-0.2	-0.1	-0.1	-0.2	-5.9	-5.8	-5.6	-5.5	-5.2	-5.1	-5.0	13.3	13.2	13.1	13.0	12.7	12.6	12.6
	6	0.001	-0.2	-0.2	-0.1	-0.1	-0.2	-0.2	-0.2	-11.7	-11.6	-11.6	-11.5	-10.9	-10.5	-10.1	16.9	16.8	16.8	16.7	16.4	16.2	16.0
	8	0.1	0.0	-0.1	0.1	-0.1	-0.1	-0.1	-0.1	-1.2	-0.8	-0.9	-0.5	-0.2	0.0	-0.1	10.3	9.9	10.0	9.7	9.6	9.4	9.5
	8	0.01	-0.2	-0.2	-0.2	-0.1	-0.2	-0.2	-0.2	-5.7	-5.8	-5.7	-5.4	-5.1	-4.9	-4.8	13.3	13.4	13.3	12.9	12.7	12.5	12.5
	8	0.001	-0.3	-0.2	-0.1	-0.2	-0.2	-0.2	-0.2	-11.7	-11.6	-11.7	-11.5	-10.9	-10.5	-10.0	17.0	16.8	16.8	16.8	16.4	16.1	15.9
