# Supplementary material for: Propensity score adjustment using machine learning classification algorithms to control selection bias in online surveys
Source: PLoS One. 2020 Apr 22;15(4):e0231500. doi: 10.1371/journal.pone.0231500 (PMC7176094; doi:10.1371/journal.pone.0231500)
Supplement: S6 Table — (PDF) [file pone.0231500.s006.pdf]

S6 Table. Mean Square Error (MSE) on the estimation of vote intention with unequal selection probabilities for the convenience sample based on the logistic formula.

			Convenience (Internet) sample sizes, in thousands of respondents																				
			Estimates for Party 1							Estimates for Party 2							Estimates for Party 3						
Method	Parameters		0.5	0.75	1	2	5	7.5	10	0.5	0.75	1	2	5	7.5	10	0.5	0.75	1	2	5	7.5	10
No adjustment			1.7	1.1	0.8	0.4	0.2	0.1	0.1	163.7	161.1	159.6	154.5	141.9	130.8	119.7	308.6	306.3	302.9	299.5	290.6	281.3	271.4
Logistic regression			3.6	2.5	1.8	1.1	0.5	0.4	0.4	10.5	7.8	6.2	3.5	1.6	1.4	1.2	112.9	109.6	107.9	103.5	100.9	100.3	99.6
C4.5	M	CP																					
	0.005	0.1	3.4	2.2	2.1	1.0	0.2	0.1	0.1	31.3	30.9	22.7	53.7	140.5	130.2	120.4	166.6	155.5	138.1	176.9	288.7	280.0	272.6
	0.005	0.25	3.5	2.6	2.1	1.2	0.2	0.1	0.1	24.7	25.9	20.5	28.6	133.0	130.5	120.1	152.6	145.5	134.0	147.4	281.5	282.1	272.5
	0.005	0.5	3.9	3.1	2.7	1.1	0.3	0.1	0.1	16.7	16.3	17.6	17.4	101.8	129.1	119.8	131.0	121.4	124.9	130.1	241.7	279.9	272.6
	0.01	0.1	3.0	2.6	2.1	0.9	0.2	0.1	0.1	30.4	32.4	24.4	58.4	140.3	131.2	119.7	172.0	156.3	137.5	181.6	289.1	282.0	272.4
	0.01	0.25	3.6	2.7	2.1	0.9	0.2	0.1	0.1	25.6	29.0	22.6	32.2	138.2	131.1	119.9	153.2	152.0	135.7	148.4	287.0	281.6	271.9
	0.01	0.5	4.3	3.2	2.3	1.2	0.2	0.1	0.1	16.6	16.8	17.5	21.2	129.6	131.2	119.7	127.3	122.3	127.9	134.6	274.9	281.8	272.5
	0.05	0.1	3.0	2.2	1.7	0.7	0.2	0.1	0.1	30.0	33.7	25.9	73.2	140.7	131.1	120.1	168.1	163.6	144.9	199.3	289.6	281.9	271.5
	0.05	0.25	3.2	2.1	1.7	0.8	0.2	0.1	0.1	28.0	28.9	26.2	48.9	141.8	130.8	119.8	162.2	153.7	146.0	167.1	290.5	282.0	272.4
	0.05	0.5	3.8	2.6	1.9	0.8	0.2	0.1	0.1	18.5	23.4	24.3	28.4	141.6	130.7	120.5	141.8	142.5	137.1	141.6	289.5	281.4	272.7
C5.0	M	CP																					
	0.005	0.1	3.0	2.1	1.9	0.7	0.2	0.1	0.1	28.3	29.9	28.4	80.8	140.2	130.5	119.6	164.4	158.0	148.9	211.3	288.5	279.9	272.5
	0.005	0.25	3.4	2.3	1.7	0.8	0.2	0.1	0.1	26.6	30.9	28.9	74.0	142.8	130.2	120.1	158.7	155.5	150.4	204.7	291.4	281.1	272.5
	0.005	0.5	3.0	2.4	1.7	0.7	0.2	0.1	0.1	28.3	28.4	31.9	74.2	141.4	131.0	119.3	162.5	153.0	150.6	201.8	290.7	280.8	272.0
	0.01	0.1	3.4	2.3	1.8	0.8	0.2	0.1	0.1	34.6	31.1	29.3	85.3	141.6	130.3	119.8	174.5	155.0	148.0	216.3	288.8	280.2	272.2
	0.01	0.25	3.3	2.3	1.6	0.8	0.2	0.1	0.1	28.1	29.1	29.6	81.3	141.7	131.0	119.7	160.6	154.3	151.6	211.5	290.7	281.1	272.9
	0.01	0.5	3.5	2.4	1.5	0.8	0.2	0.1	0.1	25.9	29.8	31.0	80.1	141.7	131.3	119.5	160.3	151.2	152.3	207.4	289.5	281.5	271.9
	0.05	0.1	3.0	2.2	1.8	0.6	0.2	0.1	0.1	29.7	29.4	28.9	95.8	141.4	130.5	119.8	171.0	157.9	149.9	226.4	289.4	280.7	272.4
	0.05	0.25	3.3	2.2	1.8	0.7	0.2	0.1	0.1	28.2	31.5	28.1	94.0	141.0	130.5	119.8	162.1	155.1	146.8	225.8	289.6	280.4	271.7
	0.05	0.5	3.1	2.2	1.5	0.7	0.2	0.1	0.1	29.3	30.0	31.7	87.6	140.5	131.3	119.8	164.3	161.9	156.9	216.9	288.4	281.7	272.6
CART	M	CP																					
	0.005	0.1	2.8	1.5	1.2	0.5	0.2	0.1	0.1	28.3	39.1	92.0	154.1	141.4	130.3	119.8	165.2	178.0	232.1	299.2	289.9	281.4	273.3
	0.005	0.25	2.9	1.2	0.9	0.4	0.2	0.1	0.1	45.0	159.0	158.6	153.6	141.8	130.8	120.1	191.7	306.0	306.9	299.8	289.4	281.2	273.1
	0.005	0.5	1.8	1.2	0.8	0.4	0.2	0.1	0.1	163.7	159.3	155.9	155.9	140.7	130.7	119.4	306.4	304.7	302.6	302.3	289.4	281.3	271.3
	0.01	0.1	2.7	1.8	1.1	0.5	0.2	0.1	0.1	28.7	38.3	95.7	153.5	140.9	130.0	119.9	169.5	177.4	232.8	301.2	289.5	280.9	272.7
	0.01	0.25	2.7	1.1	0.9	0.5	0.2	0.1	0.1	46.7	158.7	159.3	153.6	140.7	129.5	119.6	190.5	298.7	303.3	300.6	290.2	279.9	271.9
	0.01	0.5	1.7	1.3	0.9	0.4	0.2	0.1	0.1	159.8	162.3	157.6	152.7	141.5	130.6	119.8	302.9	307.4	302.3	297.9	289.1	280.8	272.2
	0.05	0.1	2.4	1.9	1.1	0.5	0.2	0.1	0.1	28.5	39.7	88.1	154.9	141.7	131.0	119.7	170.4	176.3	219.7	302.0	290.5	281.8	272.2
	0.05	0.25	2.8	1.3	0.9	0.4	0.2	0.1	0.1	49.9	159.6	160.8	154.0	141.0	131.1	119.4	192.7	303.5	306.5	300.6	288.0	281.7	270.8
	0.05	0.5	1.8	1.2	0.8	0.4	0.2	0.1	0.1	161.4	159.9	157.1	152.0	140.5	131.0	119.6	306.4	303.4	306.6	297.4	288.9	281.1	271.9
k-NN	k																						
	3		4.8	3.1	2.8	1.8	0.8	0.6	0.5	17.4	11.0	8.7	4.7	2.1	1.9	1.5	139.8	123.5	115.4	95.9	88.8	89.5	89.8
	5		4.4	3.2	2.6	1.5	0.8	0.6	0.4	14.1	9.1	7.8	4.2	2.2	1.8	1.5	124.2	104.6	106.6	91.8	85.7	86.6	86.6
	7		3.7	3.2	2.1	1.3	0.7	0.5	0.4	12.2	9.2	6.9	4.5	2.5	1.8	1.5	110.0	99.3	94.8	87.2	83.1	84.9	85.5
	9		4.1	3.2	2.2	1.4	0.7	0.5	0.4	11.5	9.3	7.4	4.1	2.4	1.8	1.4	104.1	97.4	92.3	90.1	85.0	85.6	85.4
	11		3.8	3.0	2.0	1.2	0.6	0.5	0.4	11.6	8.8	6.4	4.1	2.1	1.7	1.7	107.7	94.2	89.8	85.8	84.1	83.9	83.2
	13		4.2	2.7	2.4	1.1	0.6	0.4	0.4	11.3	9.0	6.5	3.5	2.2	1.9	1.5	105.3	95.2	89.2	88.8	84.0	84.8	85.7
Naive	laplace																						
Bayes	0		17.5	11.6	9.2	4.3	1.6	1.0	0.6	70.4	61.3	55.1	36.3	22.5	18.3	13.7	58.2	48.2	46.6	36.9	37.9	38.9	43.7
	1		20.7	12.3	9.0	3.9	1.7	1.1	0.6	75.4	58.7	48.4	36.5	23.9	15.7	13.0	64.7	47.6	48.7	35.5	35.8	41.9	44.4
	2		17.8	10.7	9.7	4.4	1.6	0.9	0.6	77.1	52.1	49.3	39.4	22.7	16.5	13.3	63.2	54.7	44.3	39.3	36.2	40.9	44.8
	5		18.4	9.4	7.0	3.5	1.6	0.9	0.6	76.8	63.0	49.8	36.5	23.0	16.5	13.5	55.4	50.2	45.3	34.6	36.9	39.9	44.6
	10		20.7	11.0	7.6	4.5	1.5	0.8	0.6	70.5	63.0	47.0	34.0	20.9	16.3	12.1	57.6	50.1	46.9	39.7	38.0	40.8	45.7
Random	mtry																						
Forest	1		11.1	6.7	6.7	8.2	8.3	5.3	2.9	28.6	55.3	68.1	102.7	60.3	22.2	9.6	76.8	40.6	30.7	26.7	41.3	68.0	108.6
	2		23.9	10.4	10.1	10.4	7.0	7.2	6.3	124.6	111.5	158.3	157.5	104.8	67.2	40.8	59.9	27.8	24.0	20.8	21.5	30.2	47.1
	4		22.3	18.7	21.3	25.3	17.8	20.0	11.9	85.4	79.7	111.2	160.1	161.7	138.4	93.9	169.1	116.8	95.8	71.9	28.3	28.3	30.0
GBM	ID	LR																					
	4	0.1	4.4	2.6	2.1	1.3	0.6	0.4	0.3	13.8	7.8	6.8	3.8	1.9	1.3	1.2	109.2	100.2	101.6	97.8	93.7	94.1	91.9
	4	0.01	2.1	1.6	1.0	0.6	0.2	0.2	0.1	39.7	35.1	36.5	34.6	31.7	29.6	28.6	184.5	173.6	177.6	172.5	167.3	163.3	162.1
	4	0.001	1.9	1.2	0.9	0.5	0.2	0.1	0.1	140.7	137.9	136.5	132.9	122.6	112.8	103.9	292.1	287.0	284.0	282.1	272.9	263.0	255.6
	6	0.1	4.2	3.2	2.4	1.3	0.6	0.5	0.3	14.1	8.5	6.6	3.6	1.8	1.5	1.4	119.4	102.6	98.6	98.0	93.1	92.0	90.9
	6	0.01	1.9	1.5	1.2	0.6	0.2	0.2	0.1	40.5	37.8	34.1	31.9	28.0	26.4	25.1	183.2	179.1	174.7	170.6	162.2	159.0	158.6
	6	0.001	1.7	1.3	0.9	0.4	0.2	0.1	0.1	140.9	136.9	137.2	133.0	119.7	111.2	102.5	291.5	286.1	285.6	278.9	270.0	262.0	255.5
	8	0.1	4.2	3.1	2.4	1.2	0.6	0.4	0.4	13.3	8.9	7.4	3.8	1.9	1.5	1.1	117.3	103.8	106.4	97.7	93.2	89.8	91.7
	8	0.01	2.3	1.6	1.1	0.6	0.2	0.2	0.1	38.5	37.4	35.5	30.9	26.6	24.3	23.1	182.3	183.0	179.3	168.6	161.5	157.0	155.6
	8	0.001	1.9	1.2	1.0	0.5	0.2	0.1	0.1	141.6	137.5	137.9	132.3	119.9	110.2	101.0	293.2	284.9	285.3	282.1	269.7	260.6	253.6
