# Supplementary material for: Propensity score adjustment using machine learning classification algorithms to control selection bias in online surveys
Source: PLoS One. 2020 Apr 22;15(4):e0231500. doi: 10.1371/journal.pone.0231500 (PMC7176094; doi:10.1371/journal.pone.0231500)
Supplement: S7 Table — (PDF) [file pone.0231500.s007.pdf]

S7 Table. Bias on the estimation of vote intention with unequal selection probabilities for the convenience sample based on the logistic formula with a sine transformation.

			Convenience (Internet) sample sizes, in thousands of respondents																				
			Estimates for Party 1							Estimates for Party 2							Estimates for Party 3						
Method	Parameters		0.5	0.75	1	2	5	7.5	10	0.5	0.75	1	2	5	7.5	10	0.5	0.75	1	2	5	7.5	10
No adjustment			-0.2	-0.3	-0.2	-0.1	-0.2	-0.2	-0.2	-7.3	-7.4	-7.3	-7.2	-7.1	-6.9	-6.7	14.5	14.7	14.5	14.4	14.3	14.2	14.1
Logistic regression			-0.1	-0.2	-0.2	0.0	-0.1	-0.1	-0.1	-0.6	-0.6	-0.4	-0.3	-0.2	-0.1	-0.2	10.2	10.3	10.0	10.0	9.9	9.9	9.9
C4.5	M	CP																					
	0.005	0.1	-0.2	-0.1	-0.1	-0.2	-0.2	-0.2	-0.2	-2.8	-2.9	-3.1	-6.9	-7.0	-6.9	-6.7	11.2	11.0	11.1	14.2	14.3	14.2	14.1
	0.005	0.25	-0.1	-0.1	-0.2	-0.1	-0.2	-0.2	-0.2	-2.2	-2.8	-2.6	-6.1	-7.0	-6.9	-6.7	10.8	10.9	10.7	13.5	14.3	14.2	14.1
	0.005	0.5	0.0	-0.2	0.2	-0.2	-0.2	-0.2	-0.2	-1.4	-2.1	-2.1	-4.0	-7.0	-6.9	-6.7	10.3	10.7	10.3	11.9	14.3	14.2	14.1
	0.01	0.1	-0.2	-0.2	-0.1	-0.2	-0.2	-0.2	-0.2	-2.8	-3.0	-3.0	-7.0	-7.0	-6.8	-6.7	11.0	11.0	11.0	14.3	14.3	14.2	14.1
	0.01	0.25	-0.3	-0.2	-0.1	-0.1	-0.2	-0.2	-0.2	-2.0	-2.8	-2.9	-6.5	-7.0	-6.9	-6.7	10.7	10.9	10.9	13.8	14.3	14.2	14.1
	0.01	0.5	-0.1	-0.3	-0.1	-0.2	-0.2	-0.2	-0.2	-1.5	-2.0	-2.4	-4.9	-7.0	-6.9	-6.7	10.1	10.5	10.7	12.6	14.3	14.2	14.1
	0.05	0.1	-0.1	-0.2	-0.1	-0.2	-0.2	-0.2	-0.2	-3.0	-3.0	-3.0	-7.1	-7.1	-6.9	-6.7	11.3	11.0	11.0	14.3	14.3	14.2	14.1
	0.05	0.25	-0.1	-0.2	-0.2	-0.2	-0.2	-0.2	-0.2	-2.3	-2.9	-2.9	-7.0	-7.0	-6.9	-6.7	10.8	11.1	10.9	14.3	14.3	14.2	14.1
	0.05	0.5	-0.1	-0.2	-0.1	-0.2	-0.1	-0.2	-0.2	-1.8	-2.5	-2.8	-6.6	-7.0	-6.9	-6.7	10.4	10.7	10.8	14.0	14.3	14.2	14.1
C5.0	M	CP																					
	0.005	0.1	-0.2	-0.2	-0.2	-0.2	-0.2	-0.2	-0.2	-2.7	-3.2	-4.0	-7.3	-7.0	-6.9	-6.7	11.1	11.4	11.8	14.5	14.3	14.2	14.1
	0.005	0.25	-0.2	-0.2	-0.2	-0.2	-0.2	-0.2	-0.2	-2.6	-3.3	-4.0	-7.2	-7.1	-6.9	-6.7	11.1	11.6	11.9	14.4	14.3	14.2	14.1
	0.005	0.5	-0.3	-0.2	-0.1	-0.2	-0.2	-0.2	-0.2	-2.4	-3.2	-4.4	-7.2	-7.0	-6.9	-6.7	10.8	11.2	12.1	14.3	14.3	14.2	14.1
	0.01	0.1	-0.3	-0.1	-0.1	-0.2	-0.2	-0.2	-0.2	-2.8	-3.2	-4.0	-7.4	-7.0	-6.9	-6.7	11.1	11.3	11.7	14.6	14.3	14.2	14.1
	0.01	0.25	-0.2	-0.1	-0.2	-0.2	-0.2	-0.2	-0.2	-2.5	-3.2	-4.3	-7.2	-7.0	-6.9	-6.7	10.9	11.3	12.0	14.4	14.4	14.2	14.1
	0.01	0.5	-0.1	0.0	-0.1	-0.2	-0.2	-0.2	-0.2	-2.5	-3.5	-4.5	-7.2	-7.0	-6.9	-6.7	10.8	11.5	12.2	14.4	14.3	14.2	14.1
	0.05	0.1	0.0	-0.2	-0.1	-0.2	-0.2	-0.2	-0.2	-2.8	-3.2	-4.0	-7.1	-7.1	-6.9	-6.7	10.9	11.2	11.7	14.3	14.4	14.2	14.1
	0.05	0.25	-0.2	-0.2	-0.2	-0.2	-0.2	-0.2	-0.2	-2.7	-3.4	-4.1	-7.2	-7.0	-6.9	-6.7	11.0	11.5	11.9	14.4	14.3	14.3	14.1
	0.05	0.5	-0.3	-0.2	-0.1	-0.2	-0.2	-0.2	-0.2	-2.8	-3.5	-4.2	-7.3	-7.0	-6.9	-6.7	11.3	11.5	12.0	14.4	14.2	14.2	14.1
CART	M	CP																					
	0.005	0.1	-0.3	-0.2	-0.2	-0.2	-0.1	-0.2	-0.2	-2.4	-4.1	-6.9	-7.3	-7.1	-6.9	-6.7	11.1	12.1	14.2	14.5	14.3	14.2	14.1
	0.005	0.25	-0.2	-0.2	-0.2	-0.1	-0.2	-0.2	-0.2	-7.2	-7.2	-7.2	-7.2	-7.0	-6.9	-6.7	14.4	14.3	14.3	14.4	14.3	14.2	14.1
	0.005	0.5	-0.1	-0.2	-0.2	-0.2	-0.2	-0.2	-0.2	-7.4	-7.4	-7.3	-7.3	-7.0	-6.9	-6.7	14.5	14.7	14.5	14.5	14.3	14.2	14.1
	0.01	0.1	-0.2	-0.1	-0.2	-0.2	-0.1	-0.2	-0.2	-2.5	-4.3	-6.9	-7.3	-7.1	-6.9	-6.7	11.0	12.2	14.2	14.4	14.3	14.2	14.1
	0.01	0.25	-0.2	-0.2	-0.2	-0.2	-0.2	-0.2	-0.2	-7.3	-7.2	-7.1	-7.2	-7.1	-6.9	-6.7	14.5	14.4	14.5	14.4	14.3	14.2	14.1
	0.01	0.5	-0.1	-0.2	-0.2	-0.2	-0.2	-0.2	-0.2	-7.2	-7.3	-7.3	-7.2	-7.0	-6.9	-6.7	14.3	14.4	14.6	14.4	14.3	14.2	14.1
	0.05	0.1	-0.3	-0.2	-0.2	-0.2	-0.2	-0.2	-0.2	-2.5	-4.5	-7.0	-7.2	-7.0	-6.9	-6.7	11.0	12.3	14.3	14.4	14.3	14.2	14.1
	0.05	0.25	-0.2	-0.1	-0.1	-0.1	-0.2	-0.2	-0.2	-7.2	-7.1	-7.2	-7.2	-7.1	-6.9	-6.7	14.4	14.2	14.3	14.4	14.3	14.2	14.1
	0.05	0.5	-0.3	-0.1	-0.2	-0.1	-0.2	-0.2	-0.2	-7.3	-7.4	-7.3	-7.3	-7.0	-6.9	-6.7	14.5	14.6	14.4	14.5	14.3	14.2	14.1
k-NN	K																						
	3		-0.1	-0.2	-0.1	-0.1	-0.1	-0.1	-0.1	-1.2	-0.6	-0.2	0.2	0.2	0.3	0.2	10.3	9.9	9.7	9.3	9.3	9.2	9.3
	5		-0.2	-0.2	-0.1	-0.2	-0.1	-0.1	-0.1	-0.3	0.1	0.2	0.5	0.4	0.3	0.4	9.9	9.5	9.2	9.2	9.2	9.2	9.1
	7		-0.1	-0.1	-0.2	-0.1	-0.2	-0.2	-0.1	0.1	0.2	0.2	0.4	0.5	0.4	0.5	9.3	9.3	9.4	9.1	9.1	9.2	9.1
	9		-0.1	-0.4	-0.2	-0.2	-0.1	-0.1	-0.1	0.0	0.3	0.4	0.5	0.6	0.4	0.4	9.4	9.4	9.3	9.1	9.0	9.2	9.2
	11		-0.1	-0.1	-0.2	-0.2	-0.1	-0.2	-0.1	0.3	0.2	0.4	0.3	0.5	0.4	0.4	9.1	9.3	9.2	9.3	9.1	9.2	9.2
	13		-0.1	-0.2	-0.1	-0.1	-0.1	-0.2	-0.1	0.0	0.4	0.4	0.5	0.4	0.4	0.4	9.4	9.1	9.2	9.1	9.1	9.1	9.2
Naive	laplace																						
Bayes	0		0.0	-0.1	-0.1	0.0	-0.1	-0.1	-0.1	1.4	1.4	1.4	1.4	1.3	1.1	0.9	8.1	8.2	8.2	8.1	8.3	8.4	8.6
	1		-0.1	-0.1	-0.1	-0.1	-0.1	-0.1	-0.1	1.7	1.4	1.5	1.4	1.1	1.1	0.9	7.9	8.0	7.9	8.2	8.4	8.5	8.6
	2		-0.1	0.0	0.0	-0.1	-0.1	-0.1	-0.1	1.5	1.6	1.6	1.5	1.2	1.1	1.0	8.1	8.0	8.0	8.2	8.4	8.5	8.6
	5		-0.1	0.0	-0.1	-0.1	-0.1	-0.1	-0.1	1.8	1.8	1.6	1.4	1.1	1.0	0.8	8.0	8.0	8.1	8.1	8.4	8.5	8.6
	10		-0.1	-0.1	-0.1	-0.1	-0.1	-0.1	-0.1	1.6	1.6	1.8	1.2	1.1	0.8	0.7	8.1	8.1	7.9	8.3	8.4	8.6	8.7
Random	mtry																						
Forest	1		-0.1	0.1	0.1	0.3	0.3	0.1	-0.1	5.7	9.6	9.7	1.4	0.8	-0.3	-2.2	5.1	1.7	1.1	6.6	8.4	9.7	11.2
	2		-0.3	0.3	0.2	0.3	0.4	0.0	0.0	9.7	11.0	10.6	6.3	4.5	3.4	1.9	2.4	0.5	0.5	3.5	5.1	6.5	7.4
	4		-0.4	0.0	0.0	0.1	0.2	0.0	-0.1	1.6	2.9	4.1	7.1	9.2	6.5	4.6	8.7	7.5	6.5	3.6	0.9	3.0	4.9
GBM	ID	LR																					
	4	0.1	-0.1	-0.2	-0.1	-0.1	-0.1	-0.1	-0.1	-0.4	-0.1	-0.2	-0.1	-0.1	-0.1	-0.1	9.7	9.6	9.5	9.4	9.5	9.5	9.5
	4	0.01	-0.2	-0.2	-0.2	-0.2	-0.2	-0.2	-0.1	-3.3	-3.4	-3.3	-3.3	-3.4	-3.3	-3.4	11.8	11.8	11.8	11.6	11.7	11.7	11.6
	4	0.001	-0.1	-0.1	-0.2	-0.1	-0.2	-0.2	-0.2	-6.7	-6.7	-6.7	-6.7	-6.5	-6.4	-6.3	14.1	14.0	14.0	14.1	13.9	13.9	13.8
	6	0.1	0.0	-0.1	0.1	-0.1	-0.1	0.0	-0.1	-0.3	-0.3	-0.3	-0.3	0.0	-0.1	0.0	9.5	9.7	9.6	9.6	9.5	9.5	9.5
	6	0.01	-0.1	-0.1	-0.1	-0.2	-0.2	-0.2	-0.2	-3.5	-3.3	-3.2	-3.2	-3.1	-3.2	-3.1	11.9	11.7	11.7	11.6	11.5	11.6	11.5
	6	0.001	-0.2	-0.2	-0.2	-0.2	-0.2	-0.2	-0.2	-6.7	-6.7	-6.8	-6.7	-6.5	-6.4	-6.2	14.1	14.1	14.0	14.1	13.9	13.8	13.8
	8	0.1	-0.2	0.0	0.0	0.0	-0.1	-0.1	-0.1	-0.5	-0.4	-0.3	0.0	0.0	0.0	-0.1	9.7	9.7	9.7	9.4	9.6	9.5	9.5
	8	0.01	-0.2	-0.2	-0.2	-0.1	-0.1	-0.2	-0.2	-3.2	-3.3	-3.3	-3.1	-3.0	-3.0	-3.0	11.9	11.8	11.7	11.6	11.5	11.5	11.5
	8	0.001	-0.2	-0.2	-0.1	-0.2	-0.2	-0.2	-0.2	-6.6	-6.7	-6.8	-6.6	-6.4	-6.4	-6.2	14.1	14.1	14.1	14.1	13.9	13.8	13.7
