# Supplementary material for: Propensity score adjustment using machine learning classification algorithms to control selection bias in online surveys
Source: PLoS One. 2020 Apr 22;15(4):e0231500. doi: 10.1371/journal.pone.0231500 (PMC7176094; doi:10.1371/journal.pone.0231500)
Supplement: S8 Table — (PDF) [file pone.0231500.s008.pdf]

S8 Table. Mean Square Error (MSE) on the estimation of vote intention with unequal selection probabilities for the convenience sample based on the logistic formula with a sine transformation.

			Convenience (Internet) sample sizes, in thousands of respondents																				
			Estimates for Party 1							Estimates for Party 2							Estimates for Party 3						
Method	Parameters		0.5	0.75	1	2	5	7.5	10	0.5	0.75	1	2	5	7.5	10	0.5	0.75	1	2	5	7.5	10
No adjustment			1.8	1.3	0.9	0.4	0.2	0.1	0.1	58.0	57.5	55.3	52.7	50.1	47.6	45.1	215.7	218.5	211.6	208.9	205.4	202.7	199.7
Logistic regression			2.9	2.0	1.4	0.9	0.5	0.3	0.3	7.5	5.7	4.0	1.9	1.1	1.0	0.8	111.4	110.9	104.2	101.1	98.6	98.7	98.8
C4.5	M	CP																					
	0.005	0.1	2.8	2.1	1.4	0.7	0.2	0.1	0.1	16.3	13.7	15.6	50.4	49.6	47.3	45.1	134.0	127.2	127.2	203.7	205.0	201.4	198.5
	0.005	0.25	3.2	2.2	1.7	0.8	0.2	0.1	0.1	13.6	13.6	11.0	43.2	49.6	47.7	45.4	124.1	123.6	119.5	185.9	204.3	202.4	199.5
	0.005	0.5	3.6	2.3	2.1	0.8	0.2	0.1	0.1	10.9	11.1	10.1	23.7	49.7	47.4	44.8	114.6	120.0	111.3	147.1	204.8	202.0	197.8
	0.01	0.1	2.7	2.1	1.5	0.6	0.2	0.1	0.1	15.3	14.3	14.2	51.8	50.0	47.1	45.1	128.2	127.3	125.6	206.1	206.2	201.3	199.3
	0.01	0.25	3.5	2.0	1.6	0.7	0.2	0.1	0.1	12.0	13.2	12.5	47.1	50.0	47.4	45.3	122.4	124.9	122.2	194.2	205.3	200.7	199.5
	0.01	0.5	3.4	2.3	1.7	0.7	0.2	0.1	0.1	12.0	10.4	10.6	32.0	49.6	47.5	45.0	113.1	115.5	118.9	162.8	204.8	201.3	199.2
	0.05	0.1	3.0	1.8	1.6	0.4	0.2	0.1	0.1	16.5	13.5	14.1	52.0	50.4	47.2	45.2	135.4	125.6	124.4	206.9	204.4	201.8	199.2
	0.05	0.25	2.8	2.0	1.5	0.5	0.2	0.1	0.1	13.9	13.9	13.7	51.2	49.9	47.4	45.5	125.9	126.7	123.8	206.6	205.1	202.6	199.1
	0.05	0.5	3.3	1.8	1.5	0.5	0.2	0.1	0.1	11.4	11.7	11.5	47.5	49.8	47.4	45.1	116.4	118.7	119.5	199.7	204.3	201.5	199.5
C5.0	M	CP																					
	0.005	0.1	2.6	1.9	1.3	0.5	0.2	0.1	0.1	14.0	16.2	23.9	54.2	49.3	48.2	45.7	130.7	135.2	144.9	211.2	205.4	202.4	199.9
	0.005	0.25	3.0	1.9	1.3	0.5	0.2	0.1	0.1	14.6	17.7	24.7	53.6	50.4	47.6	45.7	130.2	139.3	146.9	208.1	205.8	201.3	200.2
	0.005	0.5	3.1	1.8	1.4	0.5	0.2	0.1	0.1	13.0	18.7	28.5	54.2	49.0	47.6	45.4	123.6	132.0	154.3	206.6	204.0	203.2	199.7
	0.01	0.1	2.8	1.9	1.5	0.5	0.2	0.1	0.1	15.2	16.2	24.2	55.8	49.4	47.8	44.9	130.9	132.3	143.5	213.2	203.7	202.9	199.3
	0.01	0.25	2.7	1.8	1.2	0.5	0.2	0.1	0.1	13.8	17.8	27.5	53.2	49.9	47.6	45.2	127.0	133.6	150.4	208.5	206.6	202.8	199.3
	0.01	0.5	3.0	2.0	1.3	0.5	0.2	0.1	0.1	14.7	19.9	29.5	53.3	49.8	47.8	45.5	125.4	138.5	154.7	208.2	205.3	203.1	199.6
	0.05	0.1	3.3	1.8	1.3	0.5	0.2	0.1	0.1	14.9	16.1	23.6	51.6	50.3	47.8	45.6	126.8	130.0	142.4	206.1	206.5	202.1	200.1
	0.05	0.25	2.9	1.8	1.3	0.5	0.2	0.1	0.1	14.8	17.7	25.3	52.7	50.0	48.1	45.5	128.4	138.1	147.0	209.0	204.7	203.4	199.6
	0.05	0.5	2.9	1.9	1.2	0.5	0.2	0.1	0.1	16.0	18.4	26.2	53.8	49.6	47.5	45.2	134.0	138.7	150.8	209.4	203.3	202.1	198.7
CART	M	CP																					
	0.005	0.1	2.5	1.5	1.0	0.5	0.2	0.1	0.1	12.0	26.5	51.5	54.7	50.3	47.5	45.4	130.0	153.6	205.7	211.9	203.9	202.5	199.1
	0.005	0.25	1.8	1.2	0.9	0.4	0.2	0.1	0.1	56.6	53.9	53.9	52.8	49.8	47.7	45.1	211.7	208.8	208.1	207.6	205.4	202.1	199.0
	0.005	0.5	1.8	1.3	1.0	0.5	0.2	0.1	0.1	58.4	58.1	55.2	53.8	50.0	48.2	45.4	216.1	217.9	212.6	211.2	206.1	202.6	199.3
	0.01	0.1	2.4	1.3	1.1	0.4	0.2	0.1	0.1	12.6	28.3	51.6	53.7	50.2	47.1	45.1	126.9	156.5	205.7	209.2	205.5	200.5	199.2
	0.01	0.25	2.0	1.2	1.0	0.4	0.2	0.1	0.1	58.6	55.2	53.0	52.4	50.2	47.6	45.3	214.8	210.9	211.4	208.0	205.2	202.7	199.5
	0.01	0.5	1.8	1.3	1.0	0.5	0.2	0.1	0.1	56.7	55.9	55.4	52.4	49.9	47.8	45.1	210.2	210.4	214.4	208.3	204.2	201.7	199.2
	0.05	0.1	2.6	1.4	0.9	0.5	0.2	0.1	0.1	12.7	30.3	52.1	53.0	49.5	47.5	45.7	128.2	157.7	206.5	209.1	205.1	202.2	199.8
	0.05	0.25	1.8	1.1	0.8	0.4	0.2	0.1	0.1	55.7	52.9	54.4	52.7	50.1	47.5	45.4	212.8	205.8	208.1	207.9	206.2	201.7	199.9
	0.05	0.5	1.8	1.1	0.9	0.4	0.2	0.1	0.1	57.7	57.2	55.0	54.1	49.8	47.8	44.9	215.1	214.9	210.8	210.8	205.4	202.9	199.4
k-NN	k																						
	3		3.9	2.9	2.2	1.3	0.6	0.5	0.4	11.4	8.1	6.4	3.8	1.9	1.6	1.4	116.6	104.5	100.2	89.6	87.7	85.4	87.1
	5		3.4	2.7	2.2	1.2	0.6	0.5	0.4	9.9	7.1	6.3	3.7	1.9	1.7	1.5	105.9	97.3	90.5	86.7	85.0	86.3	84.4
	7		3.1	2.4	2.1	0.9	0.6	0.5	0.4	9.6	7.3	5.6	3.3	2.1	1.4	1.5	94.7	91.8	92.3	86.0	83.8	85.4	83.8
	9		3.3	2.3	1.6	1.1	0.5	0.4	0.4	8.5	6.8	5.5	3.1	2.0	1.6	1.3	96.9	93.3	90.1	84.7	81.9	85.5	85.5
	11		3.2	2.1	1.6	0.9	0.5	0.4	0.3	9.0	6.6	5.2	2.9	2.0	1.4	1.5	91.2	91.1	88.7	89.3	84.2	85.3	85.3
	13		3.6	1.9	1.5	0.9	0.5	0.4	0.4	9.4	6.8	4.9	2.7	1.8	1.6	1.4	94.8	88.4	88.9	84.7	84.5	84.0	84.9
Naive	laplace																						
Bayes	0		5.7	3.0	2.2	1.2	0.5	0.4	0.4	15.6	11.1	8.2	5.6	3.8	2.9	2.0	75.6	75.1	71.7	69.0	70.4	71.8	75.3
	1		3.9	3.3	2.1	1.1	0.5	0.4	0.4	15.2	10.1	9.1	5.3	2.9	2.7	2.2	72.8	71.4	68.5	69.9	71.7	72.5	75.4
	2		5.1	2.5	2.3	1.2	0.5	0.4	0.3	14.8	10.4	8.4	5.9	3.4	2.7	2.3	76.5	70.4	68.8	69.2	71.2	72.7	74.4
	5		5.1	2.8	2.0	1.1	0.5	0.4	0.3	16.7	11.1	8.7	5.6	3.0	2.5	2.0	74.3	70.1	70.6	69.2	71.3	73.9	75.2
	10		4.3	3.0	2.2	1.3	0.5	0.4	0.3	17.0	10.7	9.7	4.8	2.9	2.3	1.8	74.7	72.0	67.7	72.2	72.2	75.6	77.1
Random	mtry																						
Forest	1		11.0	9.0	7.6	13.2	8.0	3.8	2.2	49.4	112.1	117.0	42.7	21.4	12.1	11.6	38.9	19.6	21.4	82.3	89.3	104.8	130.2
	2		13.7	11.8	10.5	9.4	9.6	7.2	5.8	133.6	149.3	137.1	63.4	44.2	31.1	19.5	34.7	19.2	18.3	33.6	44.7	59.4	69.0
	4		27.5	23.8	15.6	10.7	10.1	12.5	11.1	87.5	72.1	80.1	86.8	115.8	76.5	51.2	144.0	107.8	94.2	41.5	25.8	35.6	49.2
GBM	ID	LR																					
	4	0.1	2.7	2.4	1.6	1.0	0.4	0.3	0.3	9.1	6.0	4.6	2.7	1.4	1.2	1.1	101.8	98.5	94.6	90.4	91.2	91.0	90.7
	4	0.01	1.8	1.4	1.1	0.5	0.2	0.1	0.1	16.5	14.5	13.8	12.1	12.0	11.7	11.7	143.7	143.2	142.8	136.0	137.4	136.9	135.8
	4	0.001	1.7	1.2	0.9	0.4	0.2	0.1	0.1	49.8	48.1	46.9	46.2	43.2	41.2	39.5	202.4	199.3	199.4	198.8	194.5	193.1	189.5
	6	0.1	3.6	2.3	1.8	1.1	0.5	0.4	0.3	8.8	6.3	4.5	2.7	1.6	1.3	1.2	98.9	100.8	96.0	94.0	91.3	90.5	90.4
	6	0.01	1.8	1.3	0.9	0.5	0.2	0.2	0.1	16.8	13.6	12.7	11.7	10.5	10.4	9.9	146.6	140.6	138.8	136.9	133.8	134.3	132.8
	6	0.001	1.9	1.2	0.9	0.5	0.2	0.1	0.1	49.0	47.9	48.0	45.6	42.1	40.9	39.1	204.6	202.5	199.7	198.7	194.5	191.6	189.4
	8	0.1	3.3	2.4	1.8	1.1	0.5	0.4	0.3	8.9	6.0	5.0	2.8	1.6	1.4	1.1	101.9	100.8	98.0	90.8	92.3	91.0	91.9
	8	0.01	2.1	1.4	1.0	0.5	0.2	0.2	0.1	14.8	13.9	13.3	11.2	9.7	9.6	9.4	145.3	142.3	140.0	136.1	132.0	132.2	131.8
	8	0.001	1.9	1.2	0.9	0.5	0.2	0.1	0.1	47.7	47.3	48.0	44.8	41.9	40.7	38.2	202.6	201.8	201.3	199.2	193.9	191.9	188.8
