# Supplementary material for: Propensity score adjustment using machine learning classification algorithms to control selection bias in online surveys
Source: PLoS One. 2020 Apr 22;15(4):e0231500. doi: 10.1371/journal.pone.0231500 (PMC7176094; doi:10.1371/journal.pone.0231500)
Supplement: S9 Table — (PDF) [file pone.0231500.s009.pdf]

S9 Table. Mean and median of bias (in absolute value) and MSE of estimates using PSA for each algorithm, and number of times its estimates have been among the best (absolute bias or MSE less than 1% greater than the minimum value)

Estimated		Bias			MSE		
party	Algorithm	Mean	Median	Best	Mean	Median	Best
Party 1	GLM	0.12	0.12	1	1.3	0.9	0
	C4.5	0.17	0.18	2	1.2	0.7	4
	C5.0	0.18	0.18	1	1.0	0.5	3
	CART	0.18	0.18	0	0.7	0.5	11
	k-NN	0.15	0.15	0	1.5	1.1	0
	Naive Bayes	0.09	0.09	10	3.1	1.3	0
	Random Forest	0.21	0.18	6	11.7	10.5	0
	GBM	0.15	0.16	1	1.0	0.6	5
Party 2	GLM	0.6	0.6	0	3.7	2.4	12
	C4.5	5.0	3.5	0	38.0	17.4	0
	C5.0	5.5	4.4	0	42.8	28.3	0
	CART	6.8	6.9	0	61.3	48.2	0
	k-NN	0.4	0.4	9	4.5	3.2	1
	Naive Bayes	2.2	1.4	0	16.5	6.9	0
	Random Forest	4.9	3.8	0	67.5	55.3	0
	GBM	3.5	2.6	13	26.6	9.3	10
Party 3	GLM	10.0	10.0	0	102.9	101.1	0
	C4.5	12.6	11.7	0	166.9	139.9	0
	C5.0	13.0	12.0	0	175.7	151.2	0
	CART	13.9	14.2	0	201.3	202.7	0
	k-NN	9.3	9.2	0	90.5	86.9	2
	Naive Bayes	8.0	8.2	0	73.2	72.0	1
	Random Forest	6.0	6.5	21	75.3	68.0	19
	GBM	11.7	11.3	0	144.9	128.2	0
Total	GLM	3.6	0.6	1	36.0	3.5	12
	C4.5	5.9	3.5	2	68.7	17.4	4
	C5.0	6.2	4.4	1	73.2	28.3	3
	CART	7.0	6.9	0	87.8	48.2	11
	k-NN	3.3	0.4	9	32.1	3.9	3
	Naive Bayes	3.4	1.4	10	30.9	10.4	1
	Random Forest	3.7	2.4	27	51.5	32.3	19
	GBM	5.1	2.6	14	57.5	9.3	15
