# Supplementary material for: Propensity score adjustment using machine learning classification algorithms to control selection bias in online surveys
Source: PLoS One. 2020 Apr 22;15(4):e0231500. doi: 10.1371/journal.pone.0231500 (PMC7176094; doi:10.1371/journal.pone.0231500)
Supplement: S10 Table — (PDF) [file pone.0231500.s010.pdf]

S10 Table. Bias and MSE of bad self-reported health prevalence estimates after reweighting with PSA using classfication algorithms.

	Bias in % for each n_{vs}					Mean Square Error for each n_{vs}				
	500	750	1000	2000	5000	500	750	1000	2000	5000
**G1 covariates**										
Logistic regression	-3.1	-3.1	-3.1	-3.1	-3.0	10.9	10.3	10.3	9.7	9.2
CART	-3.7	-3.9	-3.9	-3.9	-3.9	14.8	15.6	15.3	15.1	14.9
J48	-3.5	-3.6	-3.4	-3.8	-3.9	13.7	13.9	12.3	14.9	15.1
C5.0	-3.5	-3.5	-3.6	-3.8	-3.9	13.9	13.1	13.9	14.9	15.0
11-NN	-3.4	-3.4	-3.2	-3.3	-3.3	13.1	12.9	11.5	11.0	11.2
Nave Bayes	-3.0	-2.7	-2.7	-2.7	-2.6	14.8	34.0	28.4	16.4	10.3
Random Forest	-3.1	-3.0	-3.2	-3.3	-3.4	19.0	14.2	15.7	12.7	13.0
GBM	-3.6	-3.6	-3.6	-3.6	-3.6	14.1	13.4	13.3	12.9	12.9
**G2 covariates**										
Logistic regression	-1.2	-1.3	-1.2	-1.2	-1.1	3.7	3.5	2.8	2.4	1.9
CART	-3.3	-3.8	-3.8	-3.9	-3.9	12.5	15.1	14.9	15.3	15.0
J48	-2.5	-2.0	-1.9	-3.8	-3.9	8.8	6.3	5.6	15.0	14.9
C5.0	-2.7	-2.5	-2.9	-3.8	-3.9	9.4	8.6	10.0	15.1	15.0
11-NN	-1.8	-1.8	-1.8	-1.6	-1.7	6.2	5.2	5.0	3.8	3.7
Nave Bayes	1.0	-0.9	-1.7	-0.8	-0.7	80.7	38.2	32.2	38.9	40.8
Random Forest	-4.4	-4.1	-3.9	-3.1	-1.5	20.8	17.9	16.5	10.8	3.6
GBM	-3.1	-3.1	-3.0	-3.1	-3.0	10.9	10.2	9.6	10.0	9.0
**G3 covariates**										
Logistic regression	-2.4	-2.4	-2.4	-2.4	-2.2	8.7	7.2	6.9	6.1	5.3
CART	-3.5	-3.9	-3.8	-3.8	-3.9	13.5	15.7	14.8	14.8	15.0
J48	-3.0	-3.0	-2.8	-2.7	-3.3	10.9	10.4	9.2	8.3	11.2
C5.0	-3.1	-3.2	-3.2	-3.5	-3.9	11.3	11.0	10.9	12.7	15.0
11-NN	-3.2	-3.3	-3.3	-3.2	-3.1	12.4	12.3	12.0	10.8	9.7
Nave Bayes	-1.0	-1.0	-0.7	-0.9	-1.1	18.4	16.5	12.7	7.4	6.6
Random Forest	-3.5	-3.4	-3.3	-3.2	-3.2	13.6	12.6	12.1	10.8	10.6
GBM	-3.5	-2.7	-2.8	-3.4	-2.5	13.4	8.8	8.8	11.7	6.4
**G4 covariates**										
Logistic regression	-2.0	-2.4	-2.2	-1.6	-0.1	12.3	13.3	12.4	16.6	24.1
CART	-2.8	-3.9	-3.9	-3.9	-3.8	9.8	15.6	15.3	15.2	14.9
J48	-2.1	-2.3	-2.3	-2.2	-2.7	7.6	7.0	7.2	6.3	8.2
C5.0	-2.3	-2.8	-2.6	-3.0	-3.8	7.5	9.2	8.3	10.2	14.7
11-NN	-3.4	-3.3	-3.2	-3.1	-2.9	13.4	12.1	11.0	10.4	8.7
Nave Bayes	11.1	11.3	11.2	11.8	13.0	173.3	164.0	156.0	158.8	188.1
Random Forest	-2.9	-2.9	-2.7	-2.3	-2.1	10.2	9.7	8.3	6.3	5.2
GBM	-3.0	-2.9	-2.9	-2.9	-1.2	10.1	9.2	9.0	8.8	2.2
