# Supplementary material for: Propensity score adjustment using machine learning classification algorithms to control selection bias in online surveys
Source: PLoS One. 2020 Apr 22;15(4):e0231500. doi: 10.1371/journal.pone.0231500 (PMC7176094; doi:10.1371/journal.pone.0231500)
Supplement: S11 Table — (PDF) [file pone.0231500.s011.pdf]

S11 Table. Bias and MSE of the estimates of the percentage of households with more than two members after reweighting with PSA using classification algorithms.

	Bias in % for each n_{vs}					Mean Square Error for each n_{vs}				
	500	750	1000	2000	5000	500	750	1000	2000	5000
**G1 covariates**										
Logistic regression	10.3	10.3	10.4	10.3	10.3	109.8	108.5	110.4	107.9	107.5
CART	11.6	11.4	11.4	11.4	11.5	138.0	132.3	132.5	130.7	131.5
J48	10.5	11.3	10.7	11.4	11.4	114.8	130.1	117.2	131.4	130.1
C5.0	10.8	11.0	11.2	11.4	11.4	120.9	124.6	127.5	131.6	130.8
11-NN	10.8	10.7	10.6	10.6	10.5	120.6	118.2	115.2	113.3	110.7
Nave Bayes	10.8	10.9	10.9	10.6	10.6	136.3	126.8	124.4	115.4	113.3
Random Forest	12.4	11.8	10.3	7.9	4.9	234.5	200.3	148.1	80.7	39.7
GBM	11.0	11.0	11.0	11.0	10.9	125.2	123.3	123.4	120.9	120.0
**G2 covariates**										
Logistic regression	10.1	10.2	10.0	10.2	10.2	107.0	107.0	103.3	105.6	104.5
CART	11.3	11.5	11.4	11.3	11.4	131.6	135.7	132.5	128.9	131.3
J48	10.5	10.2	10.4	11.4	11.4	114.8	108.6	111.2	130.6	131.2
C5.0	10.3	10.7	11.1	11.5	11.4	111.9	118.1	125.6	132.2	130.9
11-NN	10.4	10.2	10.3	10.3	10.3	113.9	107.9	108.4	108.1	106.1
Nave Bayes	13.3	14.1	14.9	14.3	14.6	343.9	314.4	333.6	276.0	262.8
Random Forest	12.8	12.7	12.0	10.8	8.5	199.8	173.7	154.8	121.8	77.1
GBM	10.9	10.9	11.0	10.9	10.8	122.4	121.8	122.0	119.3	117.5
**G3 covariates**										
Logistic regression	8.2	8.1	8.0	7.8	7.7	72.6	70.2	66.6	63.0	60.8
CART	9.7	11.5	11.5	11.4	11.4	99.4	134.7	133.4	130.1	130.4
J48	8.3	8.2	8.2	8.2	9.9	74.8	71.7	71.0	69.0	101.1
C5.0	8.6	8.7	8.6	10.0	11.4	79.4	80.2	77.7	103.2	130.2
11-NN	8.8	8.6	8.6	8.7	8.8	83.2	79.2	77.7	78.0	79.0
Nave Bayes	8.1	7.7	7.8	7.3	7.2	90.8	85.8	79.4	73.9	99.0
Random Forest	10.8	10.5	10.4	10.1	9.6	122.3	114.5	112.1	104.2	95.0
GBM	10.4	8.8	8.7	9.9	7.8	112.0	81.2	79.5	99.9	62.9
**G4 covariates**										
Logistic regression	8.5	8.6	8.6	7.8	4.9	93.2	97.8	100.9	102.1	84.4
CART	9.3	11.3	11.3	11.4	11.4	92.6	129.9	128.9	131.0	131.2
J48	7.5	7.7	7.8	7.4	8.6	64.1	63.9	64.8	58.0	77.7
C5.0	7.5	8.5	8.3	9.4	11.4	63.1	77.2	73.3	92.9	130.1
11-NN	8.2	8.3	8.2	8.2	8.1	74.8	73.6	70.2	69.1	67.5
Nave Bayes	-4.8	-5.0	-5.5	-5.6	-5.0	76.1	67.4	68.9	57.3	48.3
Random Forest	9.8	9.6	9.5	8.9	8.3	101.7	95.7	92.3	81.5	72.3
GBM	9.8	9.8	9.7	9.6	6.8	100.5	99.3	96.0	94.1	48.3
