# Supplementary material for: Propensity score adjustment using machine learning classification algorithms to control selection bias in online surveys
Source: PLoS One. 2020 Apr 22;15(4):e0231500. doi: 10.1371/journal.pone.0231500 (PMC7176094; doi:10.1371/journal.pone.0231500)
Supplement: S1 Dataset — (DOCX) [file pone.0231500.s012.docx]

S1 Dataset.

The full datasets used to perform these analyses are located in

<https://osf.io/gt5mz/?view_only=162a83e57b3b43f09ec4f589599100c2>
